# Adaptive data-driven motion detection and optimized correction for brain PET

**DOI:** 10.1016/j.neuroimage.2022.119031

**Published:** 2022-03-04

**Authors:** Enette Mae Revilla, Jean-Dominique Gallezot, Mika Naganawa, Takuya Toyonaga, Kathryn Fontaine, Tim Mulnix, John A. Onofrey, Richard E. Carson, Yihuan Lu

**Affiliations:** a Department of Radiology and Biomedical Imaging, Yale University, PO Box 208048, New Haven, CT 06520-8048, USA; b Department of Urology, Yale University, New Haven, CT, USA; c Department of Biomedical Engineering, Yale University, New Haven, CT, USA

**Keywords:** PET, Head motion, Data-driven, Motion detection, Motion correction, COD

## Abstract

Head motion during PET scans causes image quality degradation, decreased concentration in regions with high uptake and incorrect outcome measures from kinetic analysis of dynamic datasets. Previously, we proposed a data-driven method, center of tracer distribution (COD), to detect head motion without an external motion tracking device. There, motion was detected using one dimension of the COD trace with a semiautomatic detection algorithm, requiring multiple user defined parameters and manual intervention. In this study, we developed a new data-driven motion detection algorithm, which is automatic, self-adaptive to noise level, does not require user-defined parameters and uses all three dimensions of the COD trace (3DCOD). 3DCOD was first validated and tested using 30 simulation studies (^18^F-FDG, *N* = 15; ^11^C-raclopride (RAC), *N* = 15) with large motion. The proposed motion correction method was tested on 22 real human datasets, with 20 acquired from a high resolution research tomograph (HRRT) scanner (^18^F-FDG, *N* = 10; ^11^C-RAC, *N* = 10) and 2 acquired from the Siemens Biograph mCT scanner. Real-time hardware-based motion tracking information (Vicra) was available for all real studies and was used as the gold standard. 3DCOD was compared to Vicra, no motion correction (NMC), one-direction COD (our previous method called 1DCOD) and two conventional frame-based image registration (FIR) algorithms, i.e., FIR1 (based on predefined frames reconstructed with attenuation correction) and FIR2 (without attenuation correction) for both simulation and real studies. For the simulation studies, 3DCOD yielded −2.3 ± 1.4% (mean ± standard deviation across all subjects and 11 brain regions) error in region of interest (ROI) uptake for ^18^F-FDG (−3.4 ± 1.7% for ^11^C-RAC across all subjects and 2 regions) as compared to Vicra (perfect correction) while NMC, FIR1, FIR2 and 1DCOD yielded −25.4 ± 11.1% (−34.5 ± 16.1% for ^11^C-RAC), −13.4 ± 3.5% (−16.1 ± 4.6%), −5.7 ± 3.6% (−8.0 ± 4.5%) and −2.6 ± 1.5% (−5.1 ± 2.7%), respectively. For real HRRT studies, 3DCOD yielded −0.3 ± 2.8% difference for ^18^F-FDG (−0.4 ± 3.2% for ^11^C-RAC) as compared to Vicra while NMC, FIR1, FIR2 and 1DCOD yielded −14.9 ± 9.0% (−24.5 ± 14.6%), −3.6 ± 4.9% (−13.4 ± 14.3%), −0.6 ± 3.4% (−6.7 ± 5.3%) and −1.5 ± 4.2% (−2.2 ± 4.1%), respectively. In summary, the proposed motion correction method yielded comparable performance to the hardware-based motion tracking method for multiple tracers, including very challenging cases with large frequent head motion, in studies performed on a non-TOF scanner.

## 1. Introduction

Head movement is a major limitation in brain positron emission tomography (PET) imaging, reducing image resolution, lowering apparent concentration in high-uptake regions, introducing attenuation-emission mismatch artifacts, and causing bias in parameter estimates fit by tracer kinetic modeling (Keller et al., 2012). In the past, many methods have been proposed to correct head motion, including frame-based image-registration (FIR) and correction using real-time hardware-based motion tracking (HMT) information (Rahmim et al., 2007; Fulton et al., 2002; Montgomery et al., 2006; Bloomfield et al., 2003; Herzog et al., 2005; Picard and Thompson, 1997; Costes et al., 2009). However, FIR cannot correct for motion within one predefined scan period (intra-frame) while HMT is not routinely used in the clinic, as setup and calibration of the tracking device can be complicated and attaching markers to each patient increases the logistical burden of the scan. Markerless motion tracking using structured light does not require any physical attachment to the patient, but its performance can be impacted by non-rigid changes in facial expression and different skin colors. Additionally, structured light approaches have not yet been fully validated (Kyme et al., 2018; Olesen et al., 2013).

Recently, several data-driven motion correction approaches (i.e., based on PET raw data itself) have been proposed. Schleyer et al. used a principal component analysis (PCA)-based method, which was first proposed in (Thielemans et al., 2013), to detect head motion with the aid of time-of-flight (TOF) information (Schleyer et al., 2015; Thielemans et al., 2013). Lu et al. proposed another data-driven algorithm, Centroid Of Distribution (COD), to detect patient motion (Lu et al., 2020; 2019b). Both PCA-based and COD-based methods used a one dimensional (1-D) PCA or COD trace, where motion detection was treated as an edge detection problem on the 1-D trace. PCA and COD traces are intrinsically noisy due to the limited count-statistics of the PET raw data. Motion detection also requires tuning multiple user-defined parameters, including the size of a median filter used to smooth the trace and a threshold for detecting motion. Therefore, these parameters control the sensitivity of motion detection, and were empirically set, rather than statistics-based, in both (Schleyer et al., 2015) and (Lu et al., 2020). Thus, these detection algorithms are tracer-distribution and count-level dependent. Note that count-level in the raw data is subject-dependent, and within the same scan, the count-level can vary greatly due to the tracer distribution change as well as the radioactive decay. Optimization of the user-defined parameters is, therefore, non-trivial. Thus, it is of interest to develop a robust data-driven motion detection approach, which does not require subject-dependent or count level-dependent parameter tuning. In (Spangler-Bickell et al., 2021), Sprangler-Bickell et al. recently performed an investigation, which focused on the proper frame duration for data-driven rigid motion estimation. Their proposed method utilized ultra-short frames for rigid motion estimation and correction. Comparably, we incorporated both short and long frames for motion estimation and correction. However, in Spangler-Bickell et al. (2021), only static data was used where tracer distribution change over time is insignificant while dynamic data sets were used in this paper. Moreover, the impact of motion correction on the accuracy of absolute quantification was not investigated.

In this study, we propose a new count statistics-based data-driven motion detection algorithm, which does not require user-parameter tuning and uses all three dimensions of the COD trace. We validated the proposed motion detection algorithm using 30 simulated 4-D (3-*D* + time) dynamic PET studies with large motion for both ^18^F-FDG and ^11^C-raclopride (^11^C-RAC) tracers. For the simulation studies, the proposed motion correction method was compared to two types of FIR algorithms and perfect motion correction, i.e., the same motion used for simulation was also used for correction (Costes et al., 2009). For real studies, the proposed motion correction method was evaluated for human dynamic scans with ^18^F-FDG (*N* = 10), ^11^C-RAC (*N* = 10), ^11^C-PBR28 (*N* = 1), and ^11^C-MRB (*N* = 1); and was compared to HMT with the Polaris Vicra tracking system (NDI Systems, Waterloo, Canada) (referred to as Vicra), which provides continuous head motion monitoring at 20 Hz and was considered as the “gold standard” (Jin et al., 2013).

## 2. Methods

### 2.1. Dynamic 4D PET simulation

#### 2.1.1. 4D digital phantom

Fifteen digital phantoms were generated based on real human PET studies, which were previously performed at the Yale PET Center. Each of the 15 subjects underwent separate PET scans with ^18^F-FDG and ^11^C-RAC. An individual attenuation map was obtained through a transmission scan before each PET scan. Each subject underwent MR scans and the individual T1-weighted MR images were segmented into 109 regions of interest (ROIs) using FreeSurfer (Fischl et al., 2004; 2002). The ROIs were resliced to the individual PET space based on the MR-PET rigid registration, which was performed using FLIRT with mutual information as the similarity metric (Jenkinson and Smith, 2001). Each individual phantom consists of 109 ROI labels in PET space (256 × 256 × 207 voxels with 1.219 × 1.219 × 1.231 mm^3^/voxel).

Motion was tracked by the Vicra system. Individual motion information, recorded at 20 Hz by the Vicra system during each real scan, was used in the simulation for the same individual phantom. The 15 subjects who underwent the largest motion magnitude were selected out of 57 examined cases for ^18^F-FDG and 143 for ^11^C-RAC. The head motion magnitude of any frame within the field-of-view (FOV) was determined from the Vicra data as twice the standard deviation of the location of eight points that were selected as the vertices of a 10-cm side-length cube centered in the scanner FOV. The final motion magnitude was the average of the values from the eight points (Jin et al., 2013).

We simulated ^18^F-FDG and ^11^C-RAC studies using the estimated kinetic parameters from two real datasets. Specifically, paired MR scans of the two real studies were segmented using FreeSurfer. For each FreeSurfer ROI, a compartmental model (the two -tissue compartment model for ^18^F-FDG, and the simplified reference tissue model for ^11^C-RAC) was used to fit the time-activity curve to generate the tracer kinetic parameters (Gallezot et al., 2020). The kinetic parameters were then used to generate noise-free time-activity curves for each ROI. The same time-activity curves were finally used for each ROI of all 15 subjects for each tracer, i.e., different brains with different motions but with the same tracer dynamics were simulated.

#### 2.1.2. Data simulation

For every simulated study, a 4-D 0–90 min dynamic simulation was performed for ^18^F-FDG (*N* = 15) and 0–60 min dynamic simulation for ^11^C-RAC (*N* = 15), in the presence of motion, to generate list-mode data. Simulations were performed for both the non-TOF Siemens HRRT and the TOF Siemens Biograph mCT scanners (Schmand et al., 1999; Jakoby et al., 2011). The list-mode TOF forward-projector model is part of the MOLAR (Motion compensation OSEM List-mode Algorithm for Resolution-Recovery Reconstruction) platform (Germino et al., 2017):

(1) $$E\left(Y_{i,t,\tau }\right)=D_{t}\left(\sum_{j}c_{i,t,j}\zeta_{i,t,\tau ,j}L_{i,t}A_{i,t}N_{i}\lambda_{j,t}\right),$$

where the system matrix element *c*_*i,t,j*_ represents the contribution from voxel *j* to the line-of-response (LOR) *i* at time *t* and accounts for scanner geometry, resolution, solid angle, and motion effects. *ζ*_*i,t,τ,j*_ is the TOF kernel, which defines the contribution of pixel *j* to TOF bin *τ* of LOR *i* at time *t*. *L*_*i,t*_ is the dimensionless product of decay factor at time *t*, live time at time *t*, and the positron branching fraction. The sensitivity normalization factor *N*_*i*_, in units of (counts/second) / (Bq/mL × mm), converts the forward projection of image *λ*_*j,t*_ (Bq/mL) to units of counts/second. *A*_*i,t*_ is the dimensionless attenuation factor. *E*(*Y*_*i,t,τ*_) is the expected number of counts in the TOF bin *τ* of LOR *i* in time bin *t*. The total scan time frame *T* (second) is divided into equal sub-bins of duration *D*_*t*_ (second) indexed by *t*. For non-TOF HRRT simulations, *ζ*_*i,t,τ,j*_ is set to 1 and the *τ* index is eliminated. Data were simulated with and without motion. Compton scatter and randoms were not simulated. Spatial resolution of 2.5 mm Full-Width-Half-Maximum (FWHM) was simulated for HRRT and 4.0 mm was simulated for mCT. TOF resolution of 580 ps in FWHM was used in the mCT simulations.

### 2.2. Real patient studies

Ten previously acquired human dynamic HRRT ^18^F-FDG (injected activity: 184 ± 4 MBq) and ten HRRT ^11^C-RAC (699 ± 66 MBq) studies were analyzed. A transmission scan was used for attenuation correction. Individual T1-weighted MR images were segmented into 109 ROIs using FreeSurfer, which were registered and resliced to the individual PET space based on the MR-PET rigid registration using mutual information (Fischl et al., 2004; 2002; Jenkinson and Smith, 2001).

Two dynamic mCT scans using ^11^C-PBR28 (0–90 min post injection) and ^11^C-MRB (0–120 min) were analyzed. ^11^C-PBR28, a PET radioligand that has high specificity for the 18-kDa translocator protein (TSPO) is used for *in vivo* measurement of neuroinflammation (Hillmer et al., 2020; Owen et al., 2014). ^11^C-MRB is used to measure brain norepinephrine transporters (NET) (Ding et al., 2010). Vicra HMT was used for motion monitoring for all the real scans for both scanners.

### 2.3. COD generation and previous detection algorithm

Head motion information was extracted from the PET list-mode data based on the COD trace (Lu et al., 2020; 2019b). To generate COD for TOF raw data, for every event *i*, the central spatial coordinate of the TOF bin, (X_*i*_, Y_*i*_, Z_*i*_), was calculated and recorded in mm from the center of the scanner FOV. The (X_*i*_, Y_*i*_, Z_*i*_) values for each event were averaged over a short time interval, e.g., 1 s, to generate raw COD traces in three directions: *C*_x_ for lateral, *C*_y_ for anterior-posterior (AP), and *C*_z_ for superior-inferior (SI) directions. For non-TOF COD, (X_*i*_, Y_*i*_, Z_*i*_) was the central coordinate of the entire LOR (Lu et al., 2020). Note that the random component of COD in each direction follows a normal distribution based on the central limit theorem.

Previously, we developed a semiautomatic algorithm to detect motion based on the COD trace, which required multiple user-defined parameters that are tracer- and noise-level dependent (Lu et al., 2020; 2019b) such as: (1) length of the median filter applied to the COD trace, which was adjusted based on empirical observation; (2) the minimum duration of the motion-free frames (MFF), which was set to 30 s (the length of the shortest dynamic frame); (3) the 5-min maximum length of an MFF as a tradeoff among the sensitivity to slow motion, the computational cost and registration accuracy and (4) the smoothing kernel for MFF reconstructions. Note that these user-defined parameters are related to motion-thresholding only. The dependency on tracer- and noise-level is further explained under the limitations in the Discussion section. After motion detection, MFF separated by detected motion time points (MTP) were reconstructed without attenuation correction (AC) and rigidly registered to a reference frame to estimate motion between each MFF and the reference frame.

### 2.4. New adaptive data-driven motion detection algorithm

**Fig. 1.**
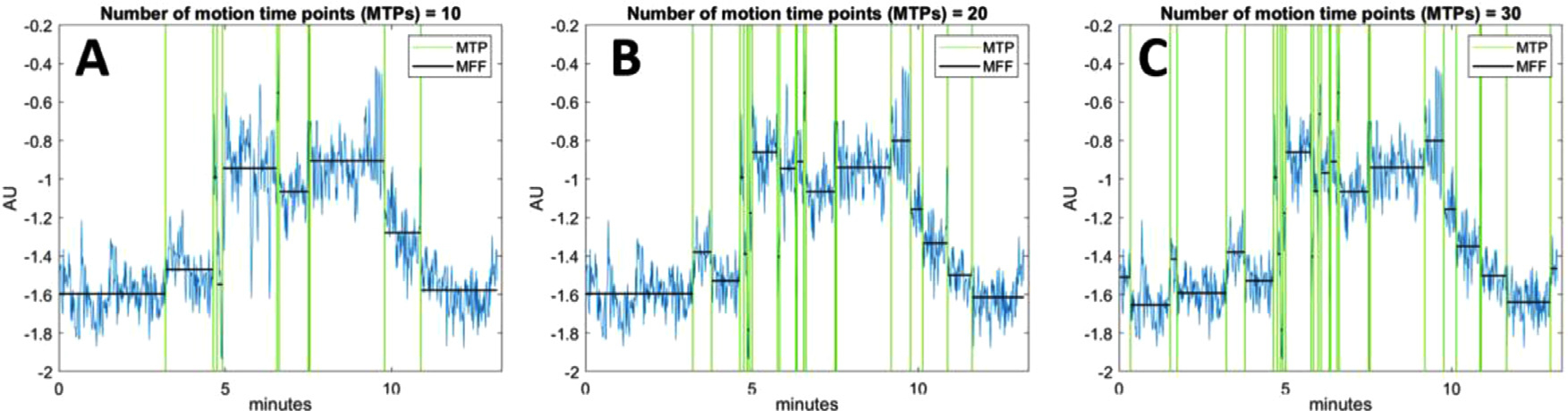
COD example (in the lateral direction) with motion detection under (A) 10 detected motion time points (MTPs, B) 20 MTPs and (C) 30 MTPs. Green vertical lines represent the MTPs and the black horizontal lines represent the mean of the COD segment within each motion-free frame (MFF), which is the period between two adjacent MTPs. AU: Arbitrary Unit.

In this study, we propose a new motion detection algorithm, which automatically detects motion based on the COD trace and is adaptive to different tracers and noise levels without user-defined parameters. First, we provide an overview of the new algorithm (Fig. 1). All the symbols used in the detection algorithm are summarized in Table 1.

Let *n* be a possible number of MTPs on the COD trace. For a given *n*, (e.g., *n* = 10) in Fig. 1A, the proposed motion detection algorithm finds the positions of all *n* MTPs that minimize an estimate of total variability within-MFF, i.e., error Fig. 1.B and C show the scenarios of *n* = 20 and 30 for the same study, respectively. The algorithm will finally choose the *n* by directly estimating the error level from the COD itself, and the corresponding MTPs are the motion detection results. We will now describe the new algorithm in detail.

#### 2.4.1. RSS error within an MFF

We will evaluate a range of *n*, and the MTPs/MFFs as follows: for a given detection result, i.e., a set of MTPs and corresponding MFFs for a *given n*, we calculate the mean of the COD trace in one direction, i.e., *C*, *within* each MFF, and compute the residual sum of squares (RSS) of the fitting. We use *C*_*m*_ to denote the COD segment within the *m*^th^ MFF. Note that the notation applies to any of the three COD directions. RSS of the *m*^th^ MFF is computed as:

(2) $${\text{RSS}}_{m}=\sum_{k}^{N_{m}}{\left(C_{m(k)}-{\bar{C}}_{m}\right)}^{2},\;\text{and}\;{\bar{C}}_{m}=\frac{1}{N_{m}}\sum_{k}^{N_{m}}C_{m(k)},$$

where *k* indexes the sampling interval, e.g., 1 s, within *C*_*m*_. *m* indexes the MFF. *N*_*m*_ is the total number of sampling intervals within the *m*^th^ MFF Eq. (2). is repeated for all *m*. By taking a sum of RSS over all the MFFs, we obtain the total residual error, i.e., *E*, of *one* detection scenario:

(3) $$E=\sum_{m}{\text{RSS}}_{m}.$$

#### 2.4.2. Choosing the MTPs for a given n using the pruned exact linear time algorithm

**Fig. 2.**
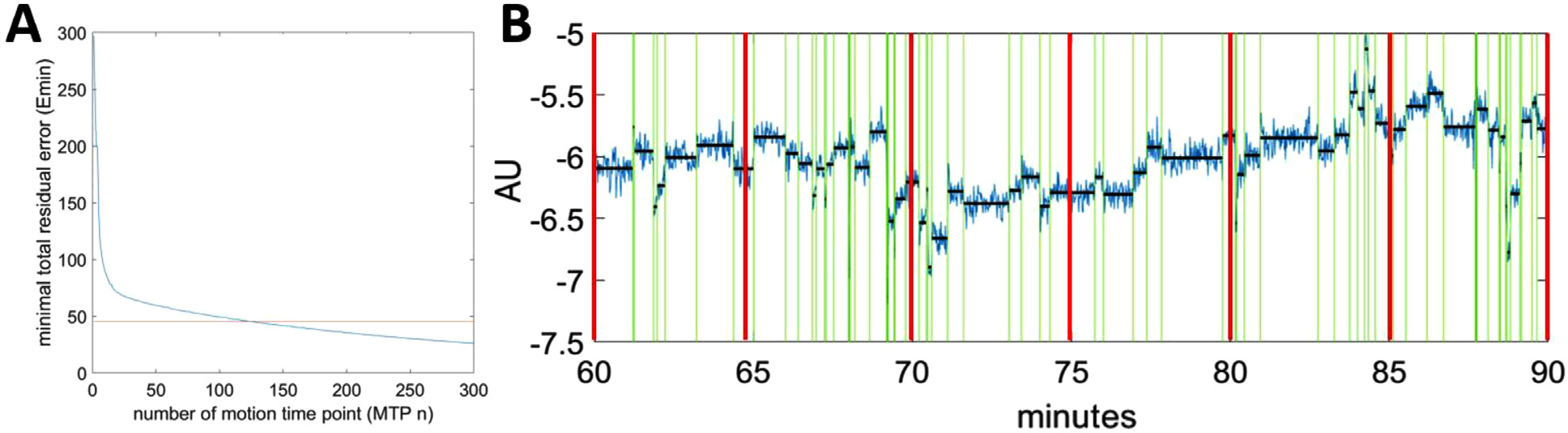
(A) *E*_min_ vs number of MTPs (*n*). The intersection between the total residual error (*E*_min_) at different MTPs and *E*_tar_ corresponds to a set of *n* MTPs. (B) An example of detection result in one COD direction (lateral) for a simulated ^18^F-FDG scan. Red vertical lines show the equal-duration partitions. The unit for this measure is arbitrary, which we denote as Arbitrary Unit (AU).

Here, we describe how to use Pruned Exact Linear Time (PELT), a changepoint detection algorithm, to find the *n* MTPs locations in the COD trace that yields the *lowest* total residual error, i.e., *E*_min_ (*n*) (Killick et al., 2012). Note that PELT guarantees finding *E*_min_ (*n*) on the condition that the noise in the COD trace is normally distributed. PELT was established based on an optimal partitioning (OP) approach, where OP belongs to one research branch in the changepoint detection literature (Jackson et al., 2005). By adding a pruning phase to the original OP algorithm, Killick et al. (2012), Jackson et al. (2005) reduced the computational cost from *O*(*T*^2^) to *O*(*T*), i.e., the computational cost linearly increases with the total data points *T*. As shown in (Killick et al., 2012), *E*_min_ (*n*) is a monotonically decreasing function of *n* (see Fig. 2A for example). Naturally, *n*(•) is a monotonically decreasing function of *E*_min_ as well. This characteristic of PELT allows us to convert the motion detection problem, i.e., finding the proper choice of *n* (and its corresponding MTPs/MFFs), to a problem of finding the proper *E*_min_, since each *E*_min_ uniquely corresponds to a set of *n* MTPs yielded by the PELT. The locations of the *n* MTPs are the motion detection results for a given *E*_min_. Note that under an extreme scenario, i.e., *n* equals the number of sampling time points in the COD trace, *E*_min_ reduces to zero. This is, of course, a meaningless detection result.

So far, we have introduced PELT, which chooses the optimal MTPs for a *given n*. Next, we will describe how to choose the desired *n*.

#### 2.4.3. Determining n

First, we apply PELT for a range of *n*, which generates an *E*_min_ vs. *n* plot (Fig. 2A), up to a predefined maximum *n*_max_, which should be higher than the most times of motion one would like to detect. In this study, we set the default *n*_max_ to 300 for a 90-min study and 200 for a 60-min study. We refer to this step as *E*_min_ scouting. Next, we find the *n* that corresponds to a target detection (*n*_tar_), which matches a unique *E* value, *E*_tar_. Ideally, *n*_tar_ and its corresponding MTPs represent the correctly detected motions based on *C*. Here, we hypothesized that the value of *E*_min_ at which head motion is accurately detected (*E*_tar_) should be approximately equal to the error for a comparable *hypothetical* scan where no motion occurred (*E*_NM_), i.e., same subject with the same injection but without motion. Note that, if no motion occurred, the variation in a COD trace would only be caused by the data noise and tracer distribution change. Of course, for a real study, it is impossible to directly obtain the *E*_NM_, since head motion will almost always occur.

Next, we show how to estimate *E*_NM_ based on the COD trace (with motion) itself, i.e., *C*. Essentially, we used partial data (without or with minimal motion) of the COD trace to estimate the total RSS contribution from data noise as well as tracer distribution change. We first divide the entire scan into several equal-duration partitions, e.g., 5 min per partition, indexed by *s*. The use of partitions makes the algorithm adaptive to the COD noise change due to isotope decay and tracer clearance. Based on the PELT detection results using *n*_max_, we use the RSS (RSS_LONG_) from the *longest P* MFFs (indexed by *p*) within partition *s* to estimate the no motion RSS for partition *s*:

(4) $${\text{RSS}}_{\text{NM},s}\approx N_{\text{PART},s}\cdot \frac{\sum_{p}{\text{RSS}}_{\text{LONG},p}}{\sum_{p}N_{\text{LONG},p}},$$

where *N*_LONG*,p*_ and *N*_PART*,s*_ are the total numbers of sampling intervals of the *p*^th^ MFF among the longest *P* MFFs and the total numbers of sampling intervals of the *s*^th^ partition, respectively (Fig. 2B). The same process is repeated for every partition. RSS_NM*,s*_ from all the partitions are summed and used as the *E*_NM_ estimate for the entire scan:

(5) $$E_{\text{NM}}\approx \sum_{s}{\text{RSS}}_{\text{NM},s}.$$

Note that if there are less than *P* MFFs in one partition, then all the MFFs inside the partition are used to estimate *E*_NM_. If an MFF *m* extends beyond one partition (*s*), the portion of the MFF *m* within the partition is used to compute RSS_NM,*s*_, i.e., ${\text{RSS}}_{\text{NM},s}=N_{\text{PART},s}\cdot \frac{{\text{RSS}}_{m}}{N_{m}}$. In this study, we set *P* equal to 2 (see Discussion).

We use the *E*_min_ vs. *n* plot to find the *n* which yields the error level *E*_tar_, and the results of PELT with *n*_tar_ shall be the final motion detection MTP set. We repeat the above detection process for all three COD directions, and a union of the MTPs from all the three directions is taken and is used as the final detection results. Note that motion detection in each direction was performed independently, so different *n*_tar_ will be different for each direction. In the validation studies (Supplementary Material), we found that there is approximately a multiplicative constant between the measured *E*_NM_ based on simulation studies and the estimated *E*_NM_ using Eq. (5). Since the measured *E*_NM_, which is referred as *E*_tar_, is what we are interested in, we introduce a constant *α* ≥ 1 to calculate the final *E*_tar_:

(6) $$E_{\text{tar}}\approx \alpha E_{\text{NM}}=\alpha \sum_{s}{\text{RSS}}_{\text{NM},\text{s}},\alpha \geq 1$$

To determine *α*, we performed comparable simulations as in the Data simulation section but *without* motion, i.e., data were simulated using the same subjects but without motion. Based on the COD trace without motion, for each study, we measured the *E*_NM_, i.e., *E*_tar_, based on the MFFs, which were determined during the detection using the COD *with* motion in the motion detection process. In other words, we applied the motion detection results, i.e., the MFFs, from COD with motion to the COD without motion, to obtain *E*_tar_. We examined different values of *α* and found that the difference (average over three directions) between estimated *E*_NM_ (Eq. (5)) and the *E*_tar_ was the smallest when *α* = 1.0 and 1.6 for ^18^F-FDG and ^11^C-RAC, respectively. Supplemental Table 1 shows the results of *E*_tar_ and *E*_NM_ for different scanners and tracers at different directions across 15 subjects. A detailed comparison of E_tar_ and E_NM_ for 5 representative studies is shown in Supplemental Table 2 for ^18^F-FDG and Supplemental Table 3 for ^11^C-RAC. Simulation studies (see Fig. 5 and Supplemental Tables 1–3) showed that *α* equals 1.0 for ^18^F-FDG, and *α* equals 1.6 for ^11^C-RAC; these values were used as the default values in the rest of the paper.

Fig. 2B shows an example of motion detection in one direction for a simulated ^18^F-FDG scan. Supplemental Fig. 1 shows the pseudo code of the entire adaptive motion detection process.

### 2.5. Final MFF determination

**Fig. 3.**
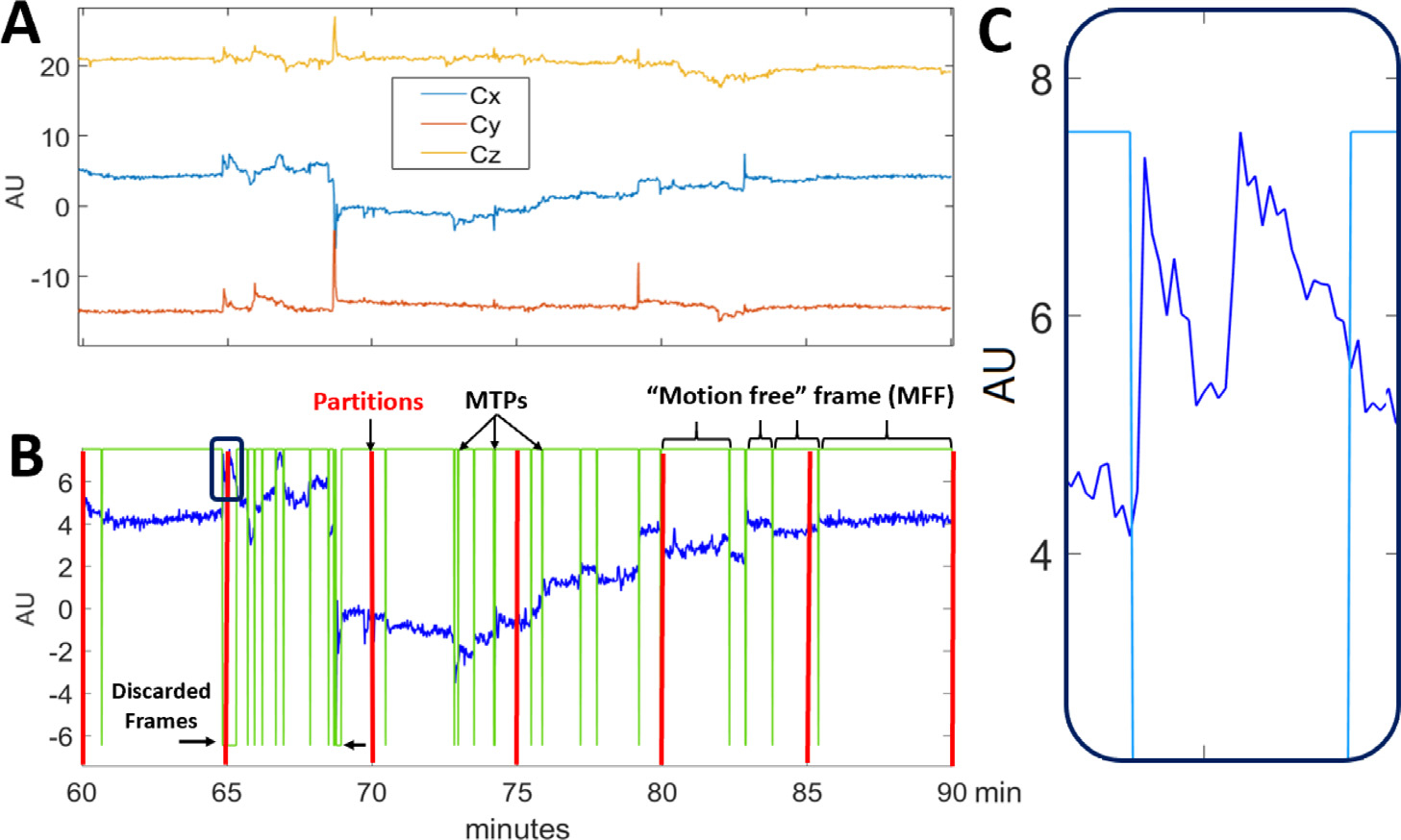
COD motion detection results for a real ^18^F-FDG study. (A) COD in lateral (*C*_x_), anterior-posterior (*C*_y_) and superior-inferior (*C*_z_) directions. (B) Example of motion detection using *C*_x_. Green vertical lines indicate motion time points (MTPs). Top horizontal line segments indicate a preserved motion-free frame (MFF) and short bottom line segments show discarded frames, where 43 s (2.4%) of the entire 30 min were discarded. Red vertical lines show the equal-duration partitions. (C) Example of a discarded frame (magnified version of black box region in (B) at 65 min) with over-frequent motion.

We observed that, after motion detection, the COD of some MFFs still contain large variations, which represent intra-frame motions and may cause inaccurate downstream motion estimation. Here, we discarded the MFFs with large intra-frame motions. Specifically, we consider an MFF to contain substantial intra-frame motions if the standard deviation of the COD trace within the MFF is larger than two times the predicted standard deviation of the partition, i.e., $2\sqrt{{\text{RSS}}_{\text{NM},s}/N_{\text{PART},s}}$, in which the MFF resides Fig. 3.A shows an example of raw COD traces in three directions: *C*_x_ for lateral, *C*_y_ for anterior-posterior and *C*_z_ for superior-inferior directions Fig. 3.B shows the detection result on *C*_x_. The green vertical lines indicate the detected MTPs. The horizontal line segments at the top of the graph indicate a preserved motion-free frame and the short line segments at the bottom of the graph indicate discarded frames due to intra-frame motions. Here, we only discard frames with large intra-frame motions while in Sec. III.B, we will show that frames with too few counts will also be discarded, since motion usually cannot be robustly estimated for those frames.

### 2.6. Optimized motion estimation and event-by-event corrected reconstruction

Motion between MFFs was estimated and corrected as follows: (1) each MFF was reconstructed using OSEM without attenuation correction; (2) the MFF image was smoothed by a *w*-mm FWHM Gaussian filter (see below for *w*), followed by a rigid registration to a 10-min post-injection reference frame; (3) a motion file consisting of all the registration transformation matrices, i.e., motion information, was built and events within one MFF were corrected using the same motion information; (4) MOLAR was used to perform event-by-event motion compensated OSEM reconstruction (2 iterations × 30 subsets for HRRT and 3 iterations × 21 subsets for mCT) with attenuation correction. Note that the final MOLAR reconstruction can be performed for any frame duration, which does not depend on the MFF timing, given its event-by-event correction nature. The discarded frames were excluded through the gating function in MOLAR. MFF registration was performed using the BioImage Suite image analysis software (Joshi et al., 2011). Details about the registration parameters can be found in the Supplemental Materials.

To quantitatively evaluate the motion estimation accuracy, we used the mean distance error (MDE, mm) for simulation studies, which is defined as follows: we first computed the center-of-mass (COM) of each FreeSurfer segmented ROI (binary map in PET space, see Supplemental Fig. 2). Ground-truth COM, i.e., COM^gt^, of each ROI was computed based on the Vicra motion information. Since Vicra motion information was used in the simulation, it provides the ground-truth motion information. The estimated location of each COM, i.e., COM^est^, based on the registration between each MFF and the reference frame for the entire scan was also computed. MDE is defined as the mean of the Euclidian distance between COM^gt^ and COM^est^ across all ROIs and over all time as:

(7) $$\text{MDE}=\frac{\sum_{t}^{N_{\text{ALL}}}\left(\frac{\sum_{i}^{N_{\text{ROI}}}\sqrt{{\left({\mathrm{COM}}_{i,t,\text{x}}^{\text{gt}}-{\mathrm{COM}}_{i,t,\text{x}}^{\text{est}}\right)}^{2}+{\left({\mathrm{COM}}_{i,t,\text{y}}^{\text{gt}}-{\mathrm{COM}}_{i,t,\text{y}}^{\text{est}}\right)}^{2}+{\left({\mathrm{COM}}_{i,t,\text{z}}^{\text{gt}}-{\mathrm{COM}}_{i,t,\text{z}}^{\text{est}}\right)}^{2}}}{N_{\text{ROI}}}\right)}{N_{\text{ALL}}}$$

*N*_ROI_ represents the number of ROI, *i* indexes the ROI; *N*_ALL_ represents the number of sampling time points of the entire scan and *t* indexes the sampling time point. Here, the COM of each ROI is moved with the ground-truth motion information as well as the estimated motion information, and MDE measures the mean distance between the two.

### 2.7. Motion correction methods for comparison

Reconstruction results based on the proposed COD-based adaptive motion detection framework (referred as “3DCOD”) were compared with two conventional FIR methods, i.e., FIR1 and FIR2 (see below), and the Vicra-based event-by-event correction. Details regarding Vicra attachment and set-up can be found in Section 2.B in (Jin et al., 2013).

In FIR1, predefined 5-min dynamic frames are first reconstructed using OSEM (2 iterations × 30 subsets) *with* AC. The same transmission-scan attenuation map is used for attenuation correction for every frame. Each frame reconstruction is smoothed by a 5-mm FWHM Gaussian filter and registered to the reference frame (10 min post injection). As a result, FIR1 suffers from both AC mismatch artifacts and intra-frame motion within each dynamic frame.

FIR2 method is similar to the 3DCOD method, but with the motion information estimated based on the predefined 5-min frames which are reconstructed *without* attenuation correction (NAC). In other words, FIR2 is the same as the 3DCOD method but with MFFs predefined as the consecutive 5-min frames. After the motion file is obtained from registration, MOLAR was used to perform motion compensated OSEM reconstruction (same MOLAR parameters as Section 2.6.) with attenuation correction. As compared to FIR1, FIR2 suffers less from the AC mismatch artifacts but still suffers from intra-frame motions. Furthermore, there would still be some misalignment from missing motion.

### 2.8. Quantitative analysis

For the simulation studies, motion detection performance was evaluated using *detectability*, which is defined as the ratio between the total number of correctly detected MTPs in all the simulated studies and the total number of ground-truth MTPs (based on the Vicra motion information) in all the simulated studies. The ground-truth MTPs were generated as follows: for each study, FreeSurfer was used to segment the paired MR into 109 ROIs (see Supplemental Fig. 2 for example), which were resliced into the individual PET space. At every time *t* (20 Hz), the brain location coordinate vector *l*_*t*_, which is defined as the average coordinate of the COMs of 8 ROIs, i.e., (left and right) frontal, occipital, hippocampus and cerebellum, was computed. *l*_*t*_ was resampled at 1 Hz by averaging over each 20 samples. Euclidian distance (Δ*l*_*t*_) between *l*_*t*_ and *l*_*t*+1_ was computed at all resampled *t*. Any *t* that yielded Δ*l*_*t*_ above a threshold (in mm) was registered as a ground-truth MTP at a given threshold. In this study, nine thresholds were evaluated: 0.5, 1, 2, 3, 4, 5, 6, 7, and 8 mm. If the detection algorithm found a MTP within 1 s before or after a ground-truth MTP, it was registered as a correctly detected MTP. Since 1 s was selected as the minimal interval for generating COD trace, we empirically allow 1 s as the threshold for accepting an MTP match to be selected. Detectability for all three COD directions, as well as using individual *C*_x_, *C*_y_, and *C*_z_, were reported.

Mean and standard deviation of the tracer uptake or standardized uptake value (SUV) percent differences compared with Vicra were computed across all the subjects for eleven FreeSurfer segmented gray-matter ROIs for ^18^F-FDG: amygdala, caudate, cerebellum cortex, frontal, hippocampus, insula, occipital, parietal, putamen, temporal and thalamus. For ^11^C-RAC, the caudate and putamen regions were used. For simulation and real studies, the proposed 3DCOD approach was compared to no motion correction (NMC), FIR1, FIR2 and Vicra. For each correction approach, the MR was registered to the 0–10 min post-injection frame of the individual method.

All the motion detection source code is available on GitHub (https://github.com/enetterevilla/COD_MotionDetection). Detailed information about the software used and computation cost can be found in the Supplemental Materials.

## 3. Results

### 3.1. Detection performance

**Fig. 4.**
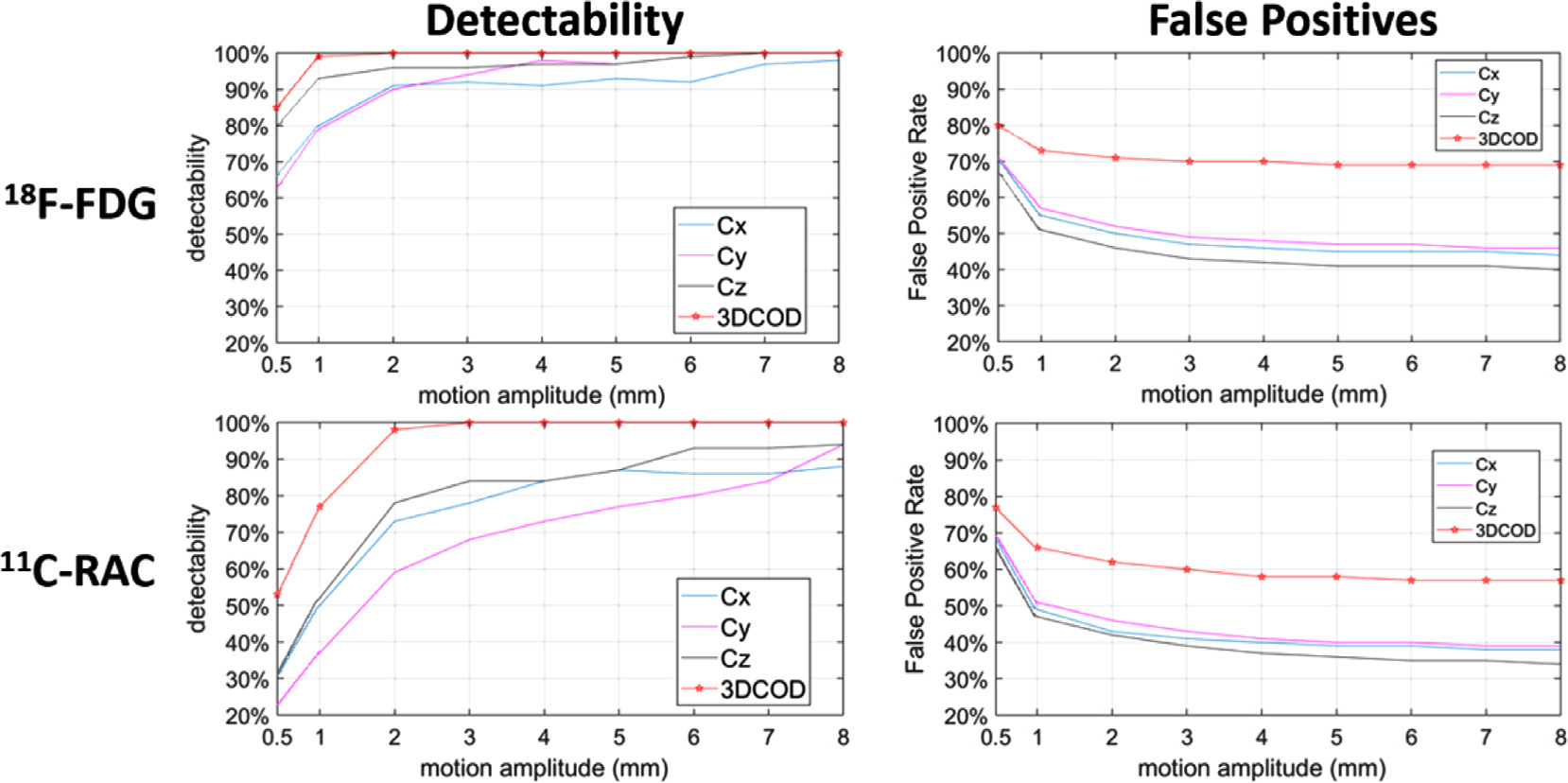
Comparison of detectability and false positive rate results between 1DCOD and 3DCOD approaches for simulated ^18^F-FDG (top) and ^11^C-RAC (bottom) HRRT studies (without TOF). Red line with stars indicates the 3DCOD method. The inflation factor *α* was set to 1.0 and 1.6 for ^18^F-FDG and ^11^C-RAC studies, respectively. Each data point in the detectability figures is the ratio between the total number of correctly detected MTPs and the total number of ground-truth MTPs in all the simulated studies at a given motion amplitude.

Fig. 4 shows the motion detectability results and false positive rates using HRRT simulation studies (^18^F-FDG studies (top) and ^11^C-RAC (bottom)) to compare motion detection based on one direction of the COD (1DCOD) trace vs. 3DCOD. The detectability was above 95% for motion above 2 mm for ^18^F-FDG and 3 mm for ^11^C-RAC, using all three directions of COD information (3DCOD, red lines). In comparison, the detectability using only one-COD direction was substantially lower for both scanners. As an example, for motion above 5 mm, the detectability of ^11^C-RAC in *C*_*y*_ only reached approximately 80% for HRRT. The detectability was lower and the false positive rate was higher for ^11^C-RAC relative to ^18^F-FDG, which was expected since ^11^C-RAC yielded noisier COD and contained more spatially centralized tracer distribution (see Supplemental Fig. 3 for example and see Discussion for details). As expected, the false positive rate decreases as the motion threshold increases. However, our proposed motion detection method showed higher false positive rate than 1DCOD since it incorporates all MTPs from all three COD directions.

**Fig. 5.**
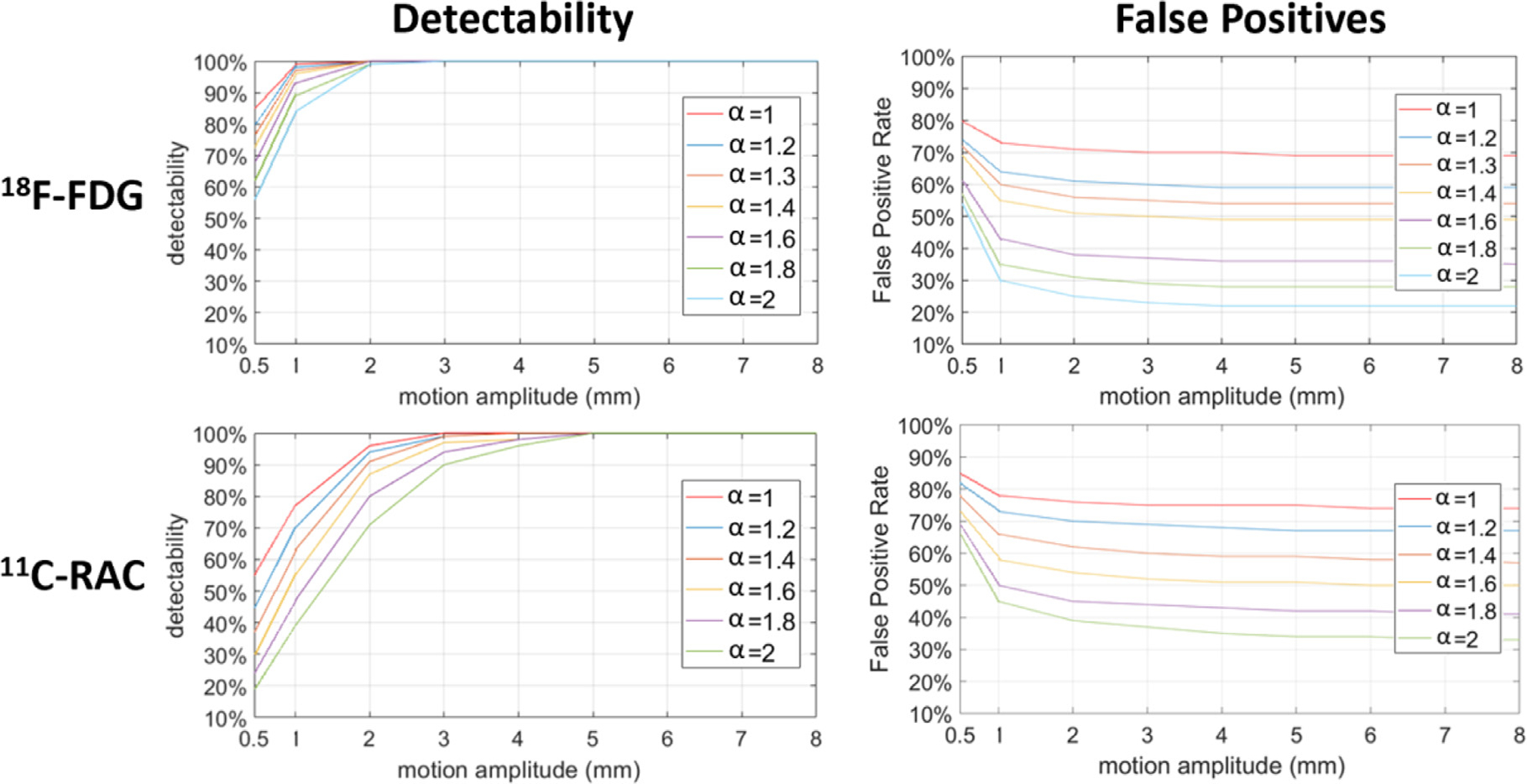
Detectability and false positive rate results for 3DCOD as a function of *α* for ^18^F-FDG (top) and ^11^C-RAC (bottom) simulated for the HRRT scanner.

Fig. 5 shows the motion detectability and false positive rates at different inflation factor *α* values (up to 2.0) for simulated HRRT ^18^F-FDG studies (top row) and ^11^C-RAC (bottom row). Practically, *α* controls the tradeoff between the motion detectability and the registration accuracy. Lower *α* leads to a more sensitive detection but also yields many short MFFs, which may hurt motion estimation accuracy due to higher noise since the motion estimation is performed by the registration between MFFs and a reference frame. In other words, we may be able to detect all the motion, but we may not be able to robustly estimate all of them. Similar to the results shown in Fig. 4, the false positive rate decreases as the *α* and the motion threshold increase.

Detectability results and false positive rates for the mCT scanner can be found in Supplemental Fig. 4 while the results at different *α* values can be found in Supplemental Fig. 5. Note that COD with TOF used the center of the TOF bin instead of the center of the LOR, which therefore provided better localization information than non-TOF (see Supplemental Fig. 6 for example). Nevertheless, the motion detection performance and false positive rate of the proposed algorithm was found to be similar between HRRT non-TOF and mCT TOF scanners.

For each tracer, we found that *α* is consistent across different subjects/scans and is also consistent between mCT and HRRT (see Supplemental Table 1). As empirically validated, *α* was set to 1.0 for ^18^F-FDG and 1.6 for ^11^C-RAC as the default in the rest of the paper. Interestingly, we found that *α* has very minimal to no effect on the PET quantification. To demonstrate this, we performed additional simulation studies by setting *α* = 2.0 for ^18^F-FDG and 3.2 for ^11^C-RAC. Supplemental Fig. 7 and Supplemental Table 4 show the reconstructed results and ROI quantification error, respectively with different *α* for FDG studies. Supplemental Fig. 8 and Supplemental Table 5 display the results for ^11^C-RAC. Results showed that there was minimal change across all regions and tracers.

### 3.2. Motion compensated reconstruction

#### 3.2.1. Simulation

Motion estimation was performed by rigid image registration using the BioImage Suite. Two similarity metrics, i.e., sum of squared differences (SSD) and mutual information (MI), were compared. Detailed results can be found in the Supplemental Materials.

**Fig. 6.**
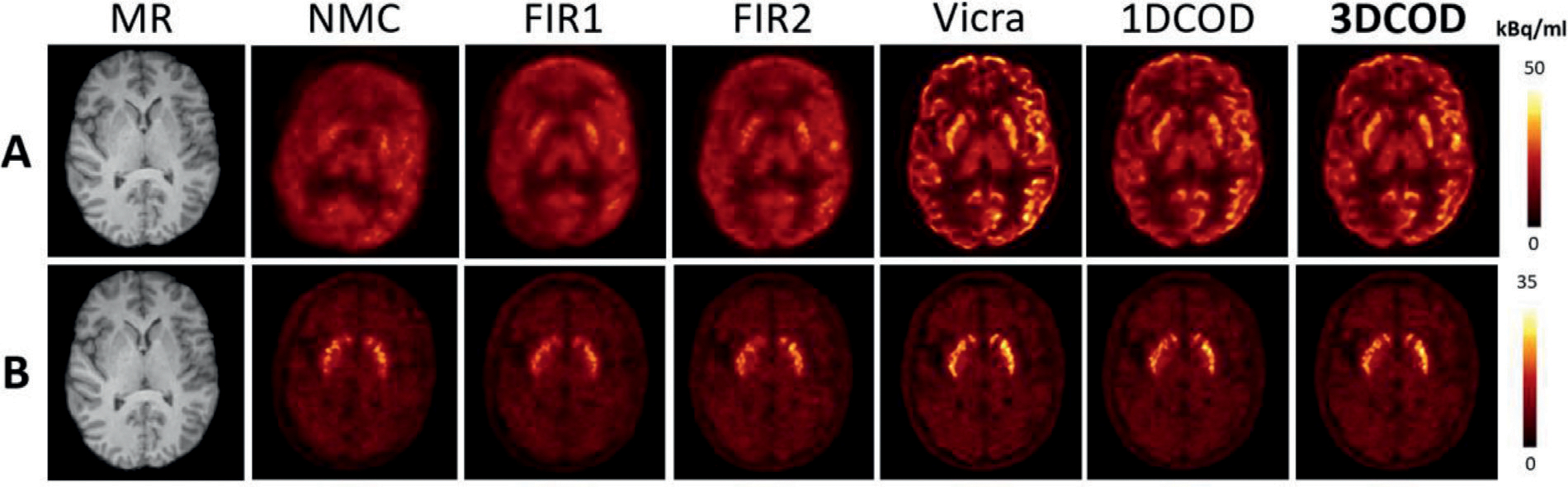
Sample slices of motion-corrected reconstructions of simulated HRRT (A) ^18^F-FDG study (60–90 min) and (B) ^11^C-RAC study (30–60 min). Studies are ranked 15/15 (worst case) based on mean difference for the 3DCOD-based approach. The best cases (1/15) can be found in Supplemental Figure 9.

In Fig. 6A, an example of reconstructed HRRT ^18^F-FDG images are shown. NMC shows very large motion which was largely corrected by 1DCOD while FIR1 and FIR2 failed to yield effective correction. 3DCOD results are slightly better than 1DCOD, with both methods restoring contrast and resolution when compared to ground truth. Quantitatively, with Vicra as the reference, the uptake error mean and standard deviation across the 11 composite brain regions (see Supplemental Table 6 and Supplemental Table 7) for this subject is −4.9 ± 3.4% for the 3DCOD method. Averaging regional errors over the 15 subjects, as shown in Table 2, NMC yielded largest mean error −25.4 % while FIR1 reduced the error to −13.4 %. FIR2 (−5.7%) provided large improvement from FIR1 whereas the 1DCOD method yielded −2.6 % mean error. The 3DCOD method produced the smallest mean error at −2.4 %, only slightly better than 1DCOD. The inter-subject variation (Average SD in Table 2), averaged over ROIs, was found to be the smallest for 3DCOD (1.4%) and 1DCOD (1.5%) as compared with FIR1 (3.5%), FIR2 (3.6%), and NMC (11.1%). The proposed method outperformed 1DCOD, specifically in the frontal region. The mean absolute error is shown in Supplemental Table 8.

In Fig. 6B, an example of ^11^C-RAC images are shown, ranking 15/15 (−6.6 ± 2.2%) in the 3DCOD method in terms of mean percent error compared to Vicra in caudate and putamen. NMC demonstrated the largest error (−34.5%), followed by the FIR1 (−16.1%), FIR2 (−8.0%) and 1DCOD (−5.1%), and the 3DCOD method provided the best result with −3.4 % error averaged over 15 subjects and both ROIs. The ROI-level mean of inter-subject deviation was smallest for 3DCOD (1.7%) as compared with the FIR1 (4.6%), FIR2 (4.5%), 1DCOD (2.7%) and NMC (16.1%) Table 3. summarizes these error results. The mean absolute error is shown in Supplemental Table 9.

For the simulated mCT studies, representatives of reconstructed ^18^F-FDG images are shown in the Supplemental Fig. 10 and quantitative results can be found in Supplemental Table 10. Both 1DCOD and 3DCOD method performed the best with −2.0 % mean error as compared with Vicra in mCT studies, followed by FIR2 (−5.8%), FIR1 (−16.7%), and NMC (−27.9%). The mean of inter-subject variation in the ROI-level showed similar trend as compared to HRRT, with the proposed motion correction method showing the least change (2.0%) compared with other methods (1DCOD: 2.1%, FIR1: 4.2%, FIR2: 3.5%, NMC: 11.5%). Two representatives of reconstructed mCT ^11^C-RAC images and quantitative results are shown in Supplemental Fig. 11 and Supplemental Table 11, respectively.

#### 3.2.2. Real patient results

**Fig. 7.**
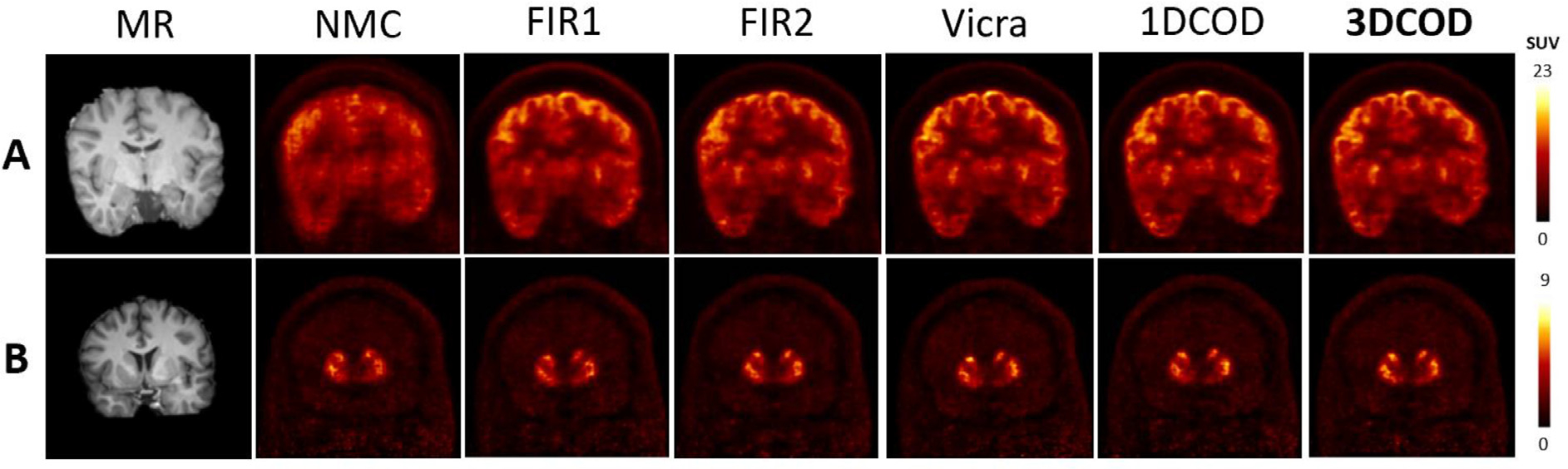
Sample slices of motion-corrected reconstructions of real (A) ^18^F-FDG (60–90 min) and (B) ^11^C-RAC (30–60 min) studies. Studies are ranked 10/10 (worst case) based on mean difference for the 3DCOD-based approach.

In Fig. 7A, an example of 60–90 min ^18^F-FDG reconstructed images are shown. Mean uptake error of 2.6 ± 4.0% (ranked 10/10, worst case), as compared to Vicra, was found for 3DCOD. The NMC image shows large head motion in the lateral direction, which was mostly corrected by the 1DCOD method. The proposed 3DCOD method further improved the results, specifically in regions like the frontal cortex and caudate. Even for the worst case scenario, the motion detection algorithm still worked robustly as shown in Table 4, which detailed the quantitative results. The proposed 3DCOD yielded minimal mean error (−0.3%) as compared with FIR1 (−3.6%), FIR2(−0.6%), 1DCOD (−1.5%) and NMC (−14.9%) across the 10 subjects, using Vicra as the reference. The ROI-level mean of inter-subject deviation was 2.8% for 3DCOD, 4.2% for 1DCOD, 4.9% for FIR1, 3.4% for FIR2 and 9.0% for NMC. For large ROIs which are prone to more motion, e.g., parietal and frontal, the 3DCOD method substantially decreased the percentage error. The mean absolute error is shown in Supplemental Table 12.

Coronal 30–60 min ^11^C-RAC reconstructed images are shown in Fig. 7B for the worst case (10/10) in the 3DCOD method in terms of mean uptake difference compared to Vicra. Quantitative results are shown in Table 5 for caudate and putamen. Across 10 subjects, NMC showed the largest difference (−24.5%) across all subjects and regions compared with FIR1 (−13.4%), FIR2 (−6.7%), 1DCOD (−2.2%) and 3DCOD (−0.4%). Additionally, the ROI-level mean of inter-subject variation with respect to Vicra was smallest for 3DCOD (3.2%) compared with FIR1 (14.3%), FIR2 (5.3%), 1DCOD (4.1%) and NMC (14.6%). At the caudate, 3DCOD reduced the error from −28.1 % (NMC) to −1.8 %. The mean absolute error is shown in Supplemental Table 13. The best cases (ranked 1/10) for both tracers can be found in Supplemental Fig. 12.

We also evaluated two studies performed in the mCT scanner to demonstrate that our proposed motion detection and correction method can work in a different scanner. Supplemental Fig. 13A shows coronal 60–90 min SUV images for an mCT ^11^C-MRB study. Detailed SUV difference results compared to Vicra are shown in Supplemental Table 14. The proposed 3DCOD method yielded smaller mean difference across all regions (2.1%), outperforming NMC (−5.1%). Supplemental Fig. 14 shows the COD traces for this subject, with large motion starting 75 min post injection in the *z* direction.

Supplemental Fig. 13B (^11^C-PBR28, mCT) shows coronal 0–90 min reconstructed images. The COD trace for this study, as shown in the Supplemental Fig. 15, indicates the subject underwent large motions in the X and Y directions for the entire scan. 3DCOD restored the image resolution. Visually, 3DCOD and Vicra yielded comparable corrected images. Quantitative results are shown in Supplemental Table 15.

## 4. Discussion

In this study, we proposed a data-driven head motion detection method that is automated and does not require user-parameter tuning (see Supplemental Table 16 for the recommended default parameters). The new algorithm automatically adapted to the noise changes in the COD for different tracers and was fast to compute (~15 s per study). Registration parameters in the motion estimation process were optimized and the motion correction was performed using event-by-event motion compensated reconstruction. The proposed motion correction method was compared with the hardware-based Vicra method, which was treated as the gold standard for real studies and ground-truth for simulations. The proposed motion correction method was also compared with two conventional frame-based image registration methods.

For both simulation and real studies, ^18^F-FDG and ^11^C-RAC were chosen to demonstrate the utility of the algorithm with respect to different tracer distributions and isotopes with different half-lives. Specifically, ^18^F-FDG is broadly distributed in the brain gray matter with longer decay time while ^11^C-RAC only contains high uptake in the subcortical region with a much shorter half-life. Image registration for ^18^F-FDG was found to be more accurate than ^11^C-RAC, as expected, since ^11^C-RAC yielded more focal distribution in the central region of the brain which provides fewer image features for image registration, as shown in the registration optimization results (Supplemental Figs. 16, 19). Note that, here, we applied a simple Gaussian filtering, albeit optimized in filter size, to reduce the noise in the MFFs before registration. In the future, we will investigate the use of deep learning-based methods (Lu et al., 2019a) to de-noise the MFFs which may improve the image registration performance.

**Fig. 8.**
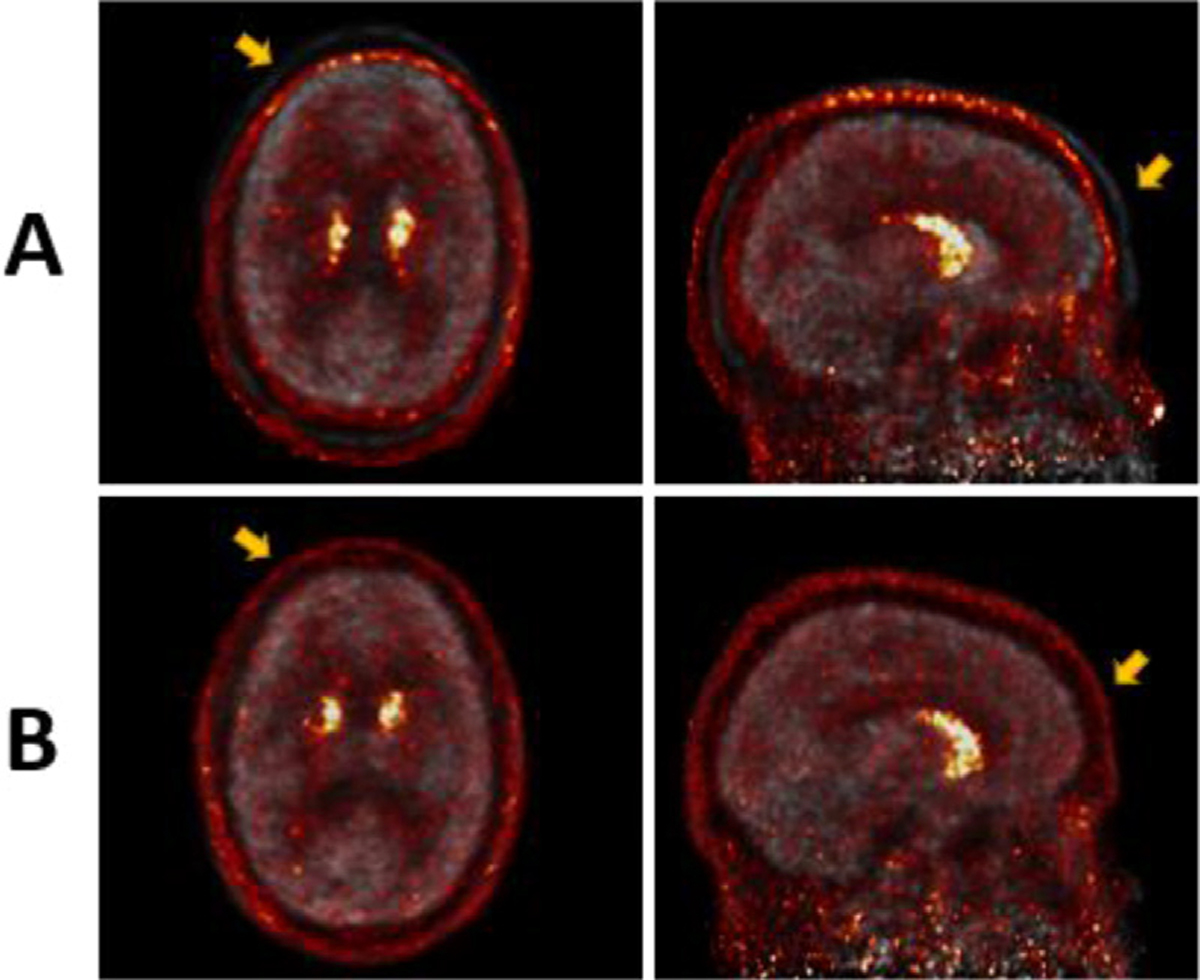
Sample slices of motion-corrected reconstructions of a HRRT ^11^C-RAC study (black and white colormap: 0–10 min, hot metal: 60–90 min) using (A) Vicra and (B) 3DCOD methods. Vicra shows a clear misalignment between the early and late frames while 3DCOD shows a good alignment.

In the simulation studies for ^18^F-FDG, parietal and frontal regions yielded relatively larger errors (~5%) for 3DCOD than other regions since both regions usually contain more motion being farther from the rotation axis of the head and are also more prone to attenuation mismatch artifacts for being adjacent to the skull. Overall, in the real studies, the 3DCOD method resulted in slightly better results, i.e., lower mean error, than the simulation studies as compared to Vicra, i.e., ^18^F-FDG (real: −0.3 ± 2.8% error, simulation: −2.4 ± 1.4%) and ^11^C-RAC (real: −0.4 ± 3.2%, simulation: −3.4 ± 1.7%). We provide three possible reasons for the differences: 1) the studies used for simulation and real studies were different, which exhibited different motion during the scans; 2) simulation is the perfect scenario but our proposed motion correction method is imperfect and still has room for improvement and 3) the real-time hardware-based motion tracking, via Vicra, may be less accurate due to large head motion and fixation issues introducing imperfect motion tracking. Vicra was used as the gold standard motion measure in the real studies, which assumed the Vicra light-reflecting tool to be firmly attached to the subject’s head. However, in practice, the tool attachment may fail, e.g., due to hair style, which causes non-rigid motion between the tool and patient head. For example, Fig. 8A shows an example of a Vicra failure case (not used in this study). After applying the Vicra-based motion correction, the mismatch between the early (black and white, 0–10 min) and the late (hot metal, 60–90 min) frames is still evident (Fig. 8A), which suggested that Vicra failed to provide reliable motion information for this study. In contrast, the proposed 3DCOD method, as shown Fig. 8B, outperformed Vicra and fixed the mismatch between the early and late frames. Note that we observed approximately 2% obvious Vicra failure instances among ~500 studies.

**Fig. 9.**
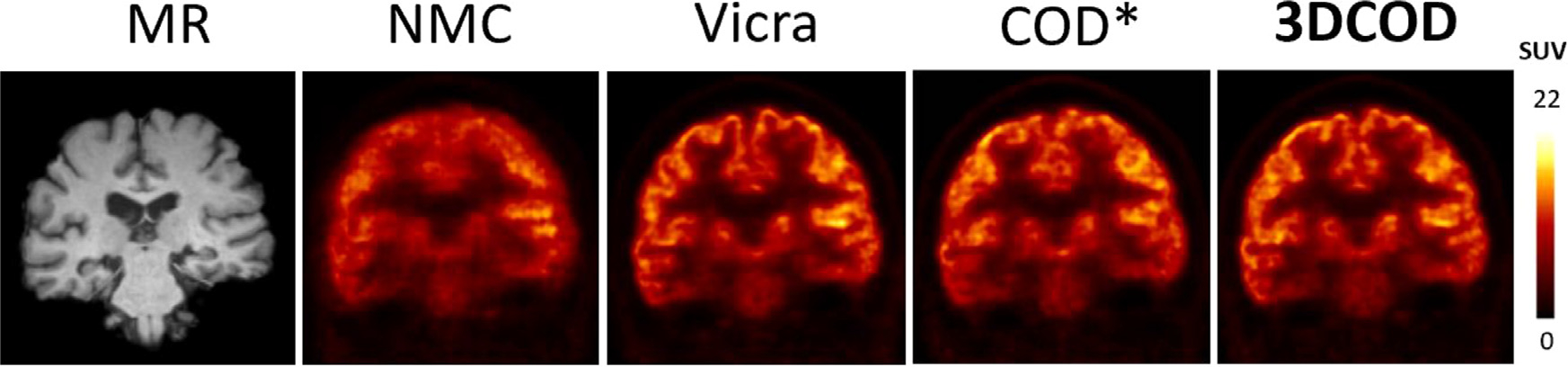
Sample slices of motion-corrected reconstructions of a real ^18^F-FDG (60–90 min) HRRT study showing the proposed 3DCOD approach compared with NMC, Vicra and COD* (example result from our previous work).

In our previous work (Lu et al., 2020), we used one dimension of the COD trace to detect and correct the motion. Across 10 subjects, the average number of MTPs using the old motion detection method is 29.3 ± 6.7. After merging the three COD axes in the proposed motion detection algorithm, the average number of MTPs significantly increased to 211.2 ± 66.9 Fig. 9. shows a representative ^18^F-FDG HRRT reconstructed images (60–90 min), comparing our proposed 3DCOD approach with the results from the motion detection method with manually selected parameters (referred to as COD*). Detailed quantitative results are shown in Table 6. The mean absolute error is shown in Supplemental Table 17. For regions like amygdala, insula, hippocampus and thalamus, approximately 5% improvement was observed for 3DCOD approach compared with COD*. For caudate, the error was reduced from −12.2 % to −2.2 % for the representative case. One of the advantages of the new approach is the utilization of three COD directions which considers all possible motions, resulting into lower mean uptake error.

As mentioned earlier, there was a significant increase in the average number of MTPs when using 3DCOD which resulted in higher false positive rates as shown in Fig. 4. False positives potentially lead to short-ened motion-free frames which can affect registration accuracy. However, undetected motion time points (false negatives) are much more likely to lead to motion artifacts in the final reconstructed images than short motion-free frames (false positives) are. Therefore, our approach is more tolerant of false positive MTPs than false negative MTPs.

The motion detection performance of the proposed algorithm was very similar for HRRT non-TOF and mCT TOF scanners. Supplemental Fig. 6 shows a COD trace for a simulated study using the same motion information and tracer but performed on different scanners. Supplemental Fig. 6B displays a somewhat noisier COD for mCT but larger separation when motion occurred. HRRT (Supplemental Fig. 6A) has a smaller separation but the motion is still distinguishable and its COD is less noisy. Note that the noise on each COD trace obeys a normal distribution when motion is absent.

A few limitations of the proposed data-driven motion detection and motion correction approach include: (1) tracer distribution change is rapid immediately after injection. As a result, COD changes quickly in response. Currently, 3DCOD approach is not reliable for motion in the early scan times, i.e., first 2 min post-injection; (2) with the increase of noise, e.g., lower dose, the motion detection accuracy will decrease and (3) our approach does not handle continuous motion perfectly. Therefore, if extremely slow motion occurs, our method may perform suboptimally.

In Spangler-Bickell et al. (2021), Sprangler-Bickell et al. recently performed a similar investigation focused on the proper frame duration for data-driven rigid motion estimation. They found that using 440 thousand true and scattered counts per frame will give a mesh error of 1 mm for ^18^F-FDG whereas we found that MFFs with more than two million counts (true coincidence) were found to provide acceptable registration accuracy, i.e., MDE < 2 mm for ^18^F-FDG. They also investigated the FWHM for smoothing and found that 4–6 mm is optimal, which is similar to our findings; 5-mm FWHM was found optimal for ^18^F-FDG while 7-mm was optimal for ^11^C-RAC for both scanners. Sprangler-Bickell et al. used two tracers with broad distribution throughout the whole brain (^18^F-FDG and ^18^F-florbetaben) while we investigated two tracers (^18^F-FDG and ^11^C-RAC) with very different brain distributions. Lastly, Sprangler-Bickell et al. did not account for intra-frame motion, which can have an impact on the motion estimation process.

Different inflation factors (*α*) were used for ^18^F-FDG (1.0) and ^11^C-RAC (1.6) in this paper. However, to further examine the robustness and the sensitivity of motion correction efficacy to the value of *α*, we tested *α*=1.6 in real ^18^F-FDG studies (data not shown), and found that the final motion correction performance, i.e., ROI quantification error, was minimally affected. This is understandable since the detection performance between *α*=1.0 and 1.6 was very small for motion larger than 1 mm (Fig. 5). Therefore, practically, for both ^18^F-FDG and ^11^C-RAC using HRRT or mCT, the same *α* choice (1.6) can be used. Other tunable parameters include *P* (number of MFFs used to predict the RSS_NM,*s*_) and the *n*_max_, which were not thoroughly tested given the high detectability of the results with these values. Although the paper only examined ^18^F-FDG and ^11^C-RAC, we also applied our motion detection method *without* parameter turning, i.e., same set of parameter for all the tracers, to many other tracers. Supplemental Fig. 20 shows examples of motion detection results for six selected tracers with a same set of parameters. In the future, we will test the algorithms for a broader range of PET tracers to further test the robustness of the algorithm.

## 5. Conclusion

We developed a new COD-based motion detection algorithm that is statistics-based, automatic and uses information from all three COD directions; thus, the algorithm is adaptive to different tracers and acquisition conditions. Preliminary results also indicate that the proposed motion detection and correction method generalize well to other radiotracers acquired with a TOF scanner. The proposed motion correction method yielded −0.3 ± 2.8% and −0.4 ± 3.2% brain region error for ^18^F-FDG and ^11^C-RAC, respectively, across 10 subjects with large head motions for each tracer, using the hardware motion tracking as reference.

## Supplementary Material

1

## Figures and Tables

**Table 1:** Definition of symbols used in the motion detection algorithm.

Symbols	Meaning

*C; C_m_; C_m(k)_*;${\bar{C}}_{m}$	COD trace in an arbitrary direction; COD segment within the *m*^th^ motion free frame (MFF); COD segment of the *k*^th^ sampling interval within the *m*^th^ MFF; averaged *C_m(k)_* over all *k*.
RSS*m*; RSS_NM,*s*_	Fitting residual sum of squares (RSS) of the *m*^th^ MFF; RSS of the partition *s* under hypothetical no-motion (NM) scenario.
*N_m_*	Total number of sampling intervals within the *m*^th^ MFF.
*N*_LONG,*p*_	Total numbers of sampling intervals of the *p*^th^ MFF among the longest MFFs within one partition.
*P*	Number of MFFs used to predict the RSS_NM,*s*_.
*N*_PART,*s*_	Total number of sampling intervals within the *s*^th^ equal-duration partition.
*E*_min_(*n*)	Lowest total residual error for *n* motion time points (MTPs) in a COD trace.
*E*_max_; *n*_tar_	Predefined maximum number of MTPs; the number of MTPs that corresponds to a target detection.
*E*_tar_; *E*_NM_	Target error level; a comparable hypothetical scan where no motion occurred *E*_NM_
α	Inflation factor
*N*_ROI_	Number of brain ROI
*N*_ALL_	Number of time sampling points of an entire scan
*l_t_*	Brain location coordinate at time *t*, defined as the average coordinate of 8 COMs of (left and right) frontal, occipital, hippocampus and cerebellum.
Δ*l_t_*	Euclidian distance between *l_t_* and *l_t+1_*.
MDE	Mean distance error
*i*	Subscript *i*, line-of-response (LOR)

**Table 2:** Mean ± Standard deviation (SD) uptake error (%) compared to Vicra for simulated ^18^ F-FDG (60–90 min) for the HRRT scanner across 15 subjects.

ROI	NMC	FIR1	FIR2	1DCOD	3DCOD

Amygdala	−13.9 ± 23.8	−3.2 ± 3.3	−0.9 ± 1.7	0.6 ± 1.9	−0.4 ± 1.7
Caudate	−31.6 ± 13.6	−9.5 ± 5.1	−7.7 ± 5.6	−2.8 ± 2.0	−2.8 ± 1.9
Cerebellum	−18.8 ± 12.1	−4.3 ± 4.2	−1.7 ± 2.1	−0.3 ± 0.9	−0.2 ± 0.7
Frontal	−42.9 ± 9.5	−13.6 ± 5.4	−12.3 ± 5.6	−6.1 ± 2.4	−5.4 ± 2.1
Hippocampus	−13.5 ± 6.9	−4.6 ± 3.4	−3.3 ± 2.7	−1.4 ± 0.8	−1.0 ± 0.8
Insula	−14.2 ± 7.3	−4.8 ± 2.8	−4.3 ± 2.5	−1.6 ± 0.8	−1.3 ± 1.1
Occipital	−24.0 ± 7.1	−10.9 ± 6.7	−6.9 ± 4.0	−4.0 ± 2.1	−4.0 ± 1.9
Parietal	−34.5 ± 10.7	−13.1 ± 5.2	−9.5 ± 4.6	−5.5 ± 2.2	−5.4 ± 2.0
Putamen	−34.0 ± 12.0	−7.4 ± 4.5	−6.0 ± 4.3	−2.4 ± 1.4	−1.9 ± 1.3
Temporal	−30.2 ± 9.6	−10.0 ± 4.9	−7.3 ± 3.8	−3.2 ± 1.4	−3.1 ± 1.3
Thalamus	−22.2 ± 9.6	−5.0 ± 2.9	−3.2 ± 2.6	−1.4 ± 0.8	−1.3 ± 0.9
Ave difference (%)	−25.4	−13.4	−5.7	−2.6	−2.3
Ave SD (%)	11.1	3.5	3.6	1.5	1.4

**Table 3:** Mean ± Standard deviation (SD) uptake error (%) compared to Vicra for simulated ^11^ C-RAC (30–60 min) for HRRT scanner across 15 subjects.

ROI	NMC	FIR1	FIR2	1DCOD	3DCOD

Caudate	−35.2 ± 15.1	−17.6 ± 5.2	−9.1 ± 5.4	−5.8 ± 3.3	−3.9 ± 2.4
Putamen	−33.8 ± 17.0	−14.6 ± 4.0	−6.9 ± 3.6	−4.4 ± 2.0	−2.9 ± 1.1
Ave difference (%)	−34.5	−16.1	−8.0	−5.1	−3.4
Ave SD (%)	16.1	4.6	4.5	2.7	1.7

**Table 4:** Mean ± Standard deviation (SD) uptake error (%) compared to Vicra for real ^18^ F-FDG (60–90 min) study on HRRT scanner across 10 subjects.

ROI	NMC	FIR1	FIR2	1DCOD	3DCOD

Amygdala	−6.9 ± 11.6	−4.0 ± 2.9	−0.3 ± 2.4	1.4 ± 6.8	0.7 ± 3.3
Caudate	−23.5 ± 10.6	−4.6 ± 7.9	−4.1 ± 5.7	−7.1 ± 6.1	−2.9 ± 4.0
Cerebellum	−10.8 ± 11.7	−4.2 ± 3.4	0.9 ± 2.5	0.9 ± 2.6	0.4 ± 2.2
Frontal	−22.9 ± 9.1	−3.1 ± 6.5	−2.9 ± 4.0	−4.0 ± 4.1	−1.6 ± 3.2
Hippocampus	−9.0 ± 4.6	−4.0 ± 2.8	−2.5 ± 1.6	−3.9 ± 3.0	−1.9 ± 3.1
Insula	−7.7 ± 5.5	−2.8 ± 3.0	−0.4 ± 1.6	1.3 ± 1.7	1.1 ± 1.5
Occipital	−11.5 ± 9.3	−1.7 ± 8.1	2.2 ± 8.0	2.1 ± 6.3	1.8 ± 5.4
Parietal	−16.5 ± 11.3	−3.2 ± 6.5	−2.4 ± 3.7	−3.7 ± 3.6	−1.5 ± 2.2
Putamen	−20.9 ± 8.7	−3.4 ± 4.8	−0.8 ± 4.2	−1.6 ± 4.0	0.2 ± 1.6
Temporal	−18.4 ± 7.7	−4.2 ± 4.3	−0.4 ± 3.2	0.6 ± 3.7	1.0 ± 2.9
Thalamus	−15.4 ± 8.7	−4.2 ± 5.0	−1.0 ± 2.3	−2.7 ± 4.0	−0.5 ± 1.8
Ave difference (%)	−14.9	−3.6	−0.6	−1.5	−0.3
Ave SD (%)	9.0	4.9	3.4	4.2	2.8

**Table 5:** Mean ± Standard deviation (SD) uptake error (%) compared to Vicra for real ^11^ C-RAC (30–60 min) study on HRRT scanner across 10 subjects.

ROI	NMC	FIR1	FIR2	1DCOD	3DCOD

Caudate	−28.1 ± 14.2	−15.4 ± 13.7	−8.5 ± 5.5	−3.9 ± 4.8	−1.8 ± 3.4
Putamen	−20.8 ± 14.9	−11.4 ± 14.8	−4.8 ± 5.0	−0.4 ± 3.4	1.0 ± 2.9
Ave difference (%)	−24.5	−13.4	−6.7	−2.2	−0.4
Ave SD (%)	14.6	14.3	5.3	4.1	3.2

**Table 6:** Mean uptake error (%) compared to Vicra for the real ^18^ F-FDG (60–90 min) study for 3DCOD and COD *.

ROI	NMC	COD*	3DCOD

Amygdala	−13.4	8.1	3.9
Caudate	−21.2	−12.2	−2.2
Cerebellum	−14.2	3.3	−0.2
Frontal	−16.3	−0.7	−1.1
Hippocampus	−5.3	6.7	−2.6
Insula	−5.8	−5.9	0.0
Occipital	−19.7	−3.2	1.6
Parietal	−12.4	−0.3	−3.4
Putamen	−19.0	−0.4	−2.8
Temporal	−18.4	−2.4	1.0
Thalamus	−11.9	−5.9	−2.6
Ave difference (%)	−14.3	−1.7	−0.8
Ave SD (%)	5.3	6.0	2.3

## Data Availability

The authors confirm that the data supporting the findings of this study are available within the article and/or its supplementary materials.
