# Supplementary material for: Adaptive data-driven motion detection and optimized correction for brain PET
Source: Neuroimage. Author manuscript; Available in PMC 2022 Jun 19. (PMC9206767; doi:10.1016/j.neuroimage.2022.119031)
Supplement: 1 [file NIHMS1794856-supplement-1.docx]

**supplemental materials**

Outline

Supplemental Table 1 - 3: *E*_NM_ and *E*_tar_ comparison

Supplemental Table 4 - 5: Uptake error using different *α*

Supplemental Table 6 - 7: Freesurfer regions

Supplemental Table 8 - 15, 17: Uptake error

Supplemental Table 16: Motion detection algorithm recommended default values

Supplemental Figure 1: Pseudo code of the motion detection algorithm

Supplemental Figure 2: Freesurfer segmented brain

Supplemental Figure 3: COD traces - ^18^F-FDG vs ^11^C-RAC

Supplemental Figure 4 - 5: Detectability and false positive results

Supplemental Figure 6: COD traces - HRRT vs mCT

Supplemental Figure 7 - 13: Reconstructed images comparison

Supplemental Figure 14 - 15: COD traces for mCT subjects

Supplemental Figure 16 - 19: Motion estimation optimization results

Supplemental Figure 20: Motion detection examples

Motion-free frame (MFF) Registration

MFF registration was performed using the BioImage Suite image analysis software. Specifically, we utilized an intensity-based image registration procedure to align images. The registration process used a gradient descent optimization algorithm to estimate the rigid transformation parameters that best align the transform image to the reference image. Our experiments leveraged 15 simulation studies for each ^18^F-FDG and ^11^C-RAC tracer, and we evaluated the optimal parameter settings for the motion estimation process using the mean distance error (MDE, see below for definition) as a figure of merit in the following fashion: 1) two different intensity similarity metrics, i.e., sum of squared difference (SSD) and mutual information (MI), were compared; 2) different resolution reduction rates were compared; where the registration algorithm utilized a multi-resolution approach to optimize the transformation from low to high image resolutions; 3) we optimized *w*, i.e., FWHM of the Gaussian smoothing kernel for the reference frame (0 and 5-mm) as well as for moving frame (0, 3, 5, 7, 9 and 11-mm); and 4) eight different count levels (~800 million count level – 100%, 50%, 20%, 10%, 5%, 2.5%, 1.25% and 0.5% counts). For all experiments, the registration algorithm utilized a hierarchical multi-resolution approach with 3 levels in the image resolution pyramid.

The reference frames used for ^18^F-FDG and ^11^C-RAC were 0-10 min post injection and 20-40 min post injection, respectively. For both HRRT and mCT, SSD (MDE_HRRT_: 1.7±0.3 mm, MDE_mCT_: 2.0±0.4 mm) was found to be superior (paired *t*-Test, *p* ≤ 0.001) in accuracy than MI (MDE_HRRT_: 2.0±0.5 mm, MDE_mCT_: 2.8±0.7 mm) for ^18^F-FDG. For ^11^C-RAC, SSD (MDE_HRRT_: 3.0±0.7 mm, MDE_mCT_: 2.3±0.3 mm) and MI (MDE_HRRT_: 3.2±0.7 mm, MDE_mCT_: 2.5±0.3 mm) yielded comparable performance (*p* > 0.01) for both scanners. Resolution rates (1.00 and 1.25) showed minor effect on the MDE. MFF smoothing with 5-mm in FWHM was found optimal for ^18^F-FDG while 7-mm was optimal for ^11^C-RAC for both scanners. We found that MFFs with more than two million counts (true coincidences) provided acceptable registration accuracy, i.e., MDE < 2 mm for ^18^F-FDG and < 4 mm for ^11^C-RAC. Therefore, after detection, MFFs with less than two million counts were discarded from further processing, i.e., discarded in both motion estimation and final reconstruction. Registration accuracy was found to be mildly dependent on the width of the post-smoothing filter for any count-level above two million. The detailed results can be found in the Supplemental Figure 11 to Supplemental Figure 14.

## **Software and computation cost**

The developed non-TOF and TOF COD generation methods were implemented in the C programming language. The COD generation for a 60-min ^18^F-FDG scan took approximately 10-20 minutes on a single-core 2.4 Hz CPU. The MATLAB R2018b function *changepoint* for the PELT algorithm implementation was used [28]. The entire detection process, including scouting and final detection across three COD directions, using a 16-core 2.1-GHz CPU, took approximately 15 seconds. Reconstruction of 300 MFFs took approximately 2 hours using 300 processors on a cluster computer. Image registration of 300 MFFs, which was performed using BioImage Suite on a 16-core 2.1-GHz CPU with 5 parallel sessions, took approximately one hour [27].

Supplemental Table 1. Mean ± Standard deviation (SD) of *E*_NM_ and *E*_tar_ for different *α* in three COD directions across 15 simulated studies. *α* = 1.0 and 1.6 are used for ^18^F-FDG and ^11^C-RAC, respectively.

|  | *C*_x_ | | *C*_y_ | | *C*_z_ | |
| --- | --- | --- | --- | --- | --- | --- |
|  | *E*_NM_ | *E*_tar_ | *E*_NM_ | *E*_tar_ | *E*_NM_ | *E*_tar_ |
| HRRT – ^18^F-FDG | 10.2±1.5 | 9.6±2.0 | 12.0±1.5 | 10.3±2.0 | 10.5±1.4 | 16.4±6.8 |
| HRRT – ^11^C-RAC | 67.4±9.8 | 63.4±12.2 | 79.2±7.0 | 76.7±10.0 | 85.5±6.5 | 91.0±22.3 |
| MCT – ^18^F-FDG | 23.5±3.0 | 25.1±4.6 | 29.7±4.4 | 28.0±4.6 | 9.2±1.5 | 13.9±5.9 |
| MCT – ^11^C-RAC | 157.9±17.9 | 161.3±22.2 | 206.2±21.5 | 194.8±24.0 | 66.9±7.8 | 72.8±17.0 |

Supplemental Table 2. Comparison of *E*_NM_ and *E*_tar_ values in three COD directions across 5 representative simulated studies using ^18^F-FDG tracer. *α* = 1.0 is used.

|  | **C*_x_*** | | **C*_y_*** | | **C*_z_*** | |
| --- | --- | --- | --- | --- | --- | --- |
| **α = 1** | **E_NM_** | **E_tar_** | **E_NM_** | **E_tar_** | **E_NM_** | **E_tar_** |
| Patient 1 | 8.6 | 7.3 | 10.8 | 8.4 | 10.5 | 9.1 |
| Patient 2 | 10.7 | 11.1 | 11.4 | 9.9 | 11.8 | 15.4 |
| Patient 3 | 8.9 | 9.0 | 11.2 | 10.1 | 10.3 | 11.9 |
| Patient 4 | 8.4 | 6.4 | 10.0 | 7.7 | 9.6 | 10.8 |
| Patient 5 | 9.1 | 9.2 | 11.1 | 10.4 | 10.3 | 14.4 |

Supplemental Table 3. Comparison of *E*_NM_ and *E*_tar_ values in three COD directions across 5 representative simulated studies using ^11^C-RAC tracer. *α* = 1.6 is used.

|  | **C*_x_*** | | **C*_y_*** | | **C*_z_*** | |
| --- | --- | --- | --- | --- | --- | --- |
| **α = 1.6** | **E_NM_** | **E_tar_** | **E_NM_** | **E_tar_** | **E_NM_** | **E_tar_** |
| Patient 1 | 55.5 | 53.2 | 72.7 | 59.6 | 83.0 | 71.5 |
| Patient 2 | 64.5 | 63.3 | 80.4 | 87.4 | 88.0 | 82.5 |
| Patient 3 | 63.2 | 62.6 | 73.2 | 73.3 | 87.3 | 85.5 |
| Patient 4 | 51.8 | 48.7 | 68.0 | 73.1 | 87.2 | 74.8 |
| Patient 5 | 61.1 | 58.8 | 74.3 | 72.9 | 79.2 | 83.6 |

Supplemental Table 4. Mean ± Standard deviation (SD) uptake error (%) compared to Vicra for simulated ^18^F-FDG (60-90 minutes) for the HRRT scanner across 15 subjects using different α.

| **ROI** | **3DCOD**  **α = 1** | **3DCOD**  **α = 2** |
| --- | --- | --- |
| Amygdala | -0.4±1.7 | 0.3±1.5 |
| Caudate | -2.8±1.9 | -2.6±2.0 |
| Cerebellum | -0.2±0.7 | -0.2±0.8 |
| Frontal | -5.4±2.1 | -5.5±2.3 |
| Hippocampus | -1.0±0.8 | -1.2±1.0 |
| Insula | -1.3±1.1 | -1.4±0.9 |
| Occipital | -4.0±1.9 | -3.9±1.9 |
| Parietal | -5.4±2.0 | -5.2±2.2 |
| Putamen | -1.9±1.3 | -1.7±1.3 |
| Temporal | -3.1±1.3 | -3.0±1.4 |
| Thalamus | -1.3±0.9 | -1.5±0.9 |
| **Ave difference (%)** | -2.3 | -2.3 |
| **Ave SD (%)** | 1.4 | 1.5 |

Supplemental Table 5. Mean ± Standard deviation (SD) uptake error (%) compared to Vicra for simulated ^11^C-RAC (30-60 minutes) for the HRRT scanner across 15 subjects using different α.

| **ROI** | **3DCOD**  **α = 1.6** | **3DCOD**  **α = 3.2** |
| --- | --- | --- |
| Caudate | -3.9±2.4 | -4.0±2.3 |
| Putamen | -2.9±1.1 | -3.1±1.1 |
| **Ave difference (%)** | -3.4 | -3.6 |
| **Ave SD (%)** | 1.7 | 1.7 |

Supplemental Table 6. Regions with corresponding Freesurfer label names and label numbers.

|  | **Right Hemisphere** | | **Left Hemisphere** | | |
| --- | --- | --- | --- | --- | --- |
| **Region** | **Label Name** | **Label Number** | **Label Name** | **Label Number** |  |
| Frontal pole | ctx-rh-frontalpole | 2032 | ctx-lh-frontalpole | 1032 |  |
| Superior frontal gyrus | ctx-rh-superiorfrontal | 2028 | ctx-lh-superiorfrontal | 1028 |  |
| Rostral middle frontal gyrus | ctx-rh-rostralmiddlefrontal | 2027 | ctx-lh-rostralmiddlefrontal | 1027 |  |
| Caudal middle frontal gyrus | ctx-rh-caudalmiddlefrontal | 2003 | ctx-lh-caudalmiddlefrontal | 1003 |  |
| Pars orbitals | ctx-rh-parsorbitalis | 2019 | ctx-lh-parsorbitalis | 1019 |  |
| Pars opercularis | ctx-rh-parsopercularis | 2018 | ctx-lh-parsopercularis | 1018 |  |
| Pars triangularis | ctx-rh-parstriangularis | 2020 | ctx-lh-parstriangularis | 1020 |  |
| Lateral orbitofrontal | ctx-rh-lateralorbitofrontal | 2012 | ctx-lh-lateralorbitofrontal | 1012 |  |
| Medal orbitofrontal | ctx-rh-medialorbitofrontal | 2014 | ctx-lh-medialorbitofrontal | 1014 |  |
| Temporal pole | ctx-rh-temporalpole | 2033 | ctx-lh-temporalpole | 1033 |  |
| Entorhinal cortex | ctx-rh-entorhinal | 2006 | ctx-lh-entorhinal | 1006 |  |
| Parahippocampal cortex | ctx-rh-parahippocampal | 2016 | ctx-lh-parahippocampal | 1016 |  |
| Hippocampus | Right-Hippocampus | 53 | Left-Hippocampus | 17 |  |
| Amygdala | Right-Amygdala | 54 | Left-Amygdala | 18 |  |
| Inferior temporal gyrus | ctx-rh-inferiortemporal | 2009 | ctx-lh-inferiortemporal | 1009 |  |
| Fusiform gyrus | ctx-rh-fusiform | 2007 | ctx-lh-fusiform | 1007 |  |
| Middle temporal gyrus | ctx-rh-middletemporal | 2015 | ctx-lh-middletemporal | 1015 |  |
| Banks of the superior temporal sulcus | ctx-rh-bankssts | 2001 | ctx-lh-bankssts | 1001 |  |
| Superior temporal gyrus | ctx-rh-superiortemporal | 2030 | ctx-lh-superiortemporal | 1030 |  |
| Transverse temporal gyrus | ctx-rh-transversetemporal | 2034 | ctx-lh-transversetemporal | 1034 |  |
| Supramarginal gyrus | ctx-rh-supramarginal | 2031 | ctx-lh-supramarginal | 1031 |  |
| Insular cortex | ctx-rh-insula | 2035 | ctx-lh-insula | 1035 |  |
| Precuneus | ctx-rh-precuneus | 2025 | ctx-lh-precuneus | 1025 |  |
| Paracentrual gyrus | ctx-rh-paracentral | 2017 | ctx-lh-paracentral | 1017 |  |
| Postcentral gyrus | ctx-rh-postcentral | 2022 | ctx-lh-postcentral | 1022 |  |
| Precentral gyrus | ctx-rh-precentral | 2024 | ctx-lh-precentral | 1024 |  |
| Superior parietal lobule | ctx-rh-superiorparietal | 2029 | ctx-lh-superiorparietal | 1029 |  |
| Inferior parietal lobule | ctx-rh-inferiorparietal | 2008 | ctx-lh-inferiorparietal | 1008 |  |
| Lateral occipital cortex | ctx-rh-lateraloccipital | 2011 | ctx-lh-lateraloccipital | 1011 |  |
| Cuneus | ctx-rh-cuneus | 2005 | ctx-lh-cuneus | 1005 |  |
| Pericalcarine cortex | ctx-rh-pericalcarine | 2021 | ctx-lh-pericalcarine | 1021 |  |
| Lingual gyrus | ctx-rh-lingual | 2013 | ctx-lh-lingual | 1013 |  |
| Cuadate | Right-Caudate | 50 | Left-Caudate | 11 |  |
| Putamen | Right-Putamen | 51 | Left-Putamen | 12 |  |
| Thalamus | Right-Thalamus-Proper | 49 | Left-Thalamus-Proper | 10 |  |
| Cerebellum Cortex | Right-Cerebellum-Cortex | 47 | Left-Cerebellum-Cortex | 8 |  |

Label names and numbers are from the Freesurfer 6.0 Desikan-Killiany atlas and color look up table.

**Supplemental Table 7**. Composition of composite regions.

| **Composite Region** | **Individual Region** | **Right Label Number** | **Left Label Number** |
| --- | --- | --- | --- |
| **Frontal** | Frontal pole | 2032 | 1032 |
|  | Superior frontal gyrus | 2028 | 1028 |
|  | Pars orbitals | 2019 | 1019 |
|  | Pars opercularis | 2018 | 1018 |
|  | Pars triangularis | 2020 | 1020 |
|  | Lateral orbitofrontal | 2012 | 1012 |
|  | Medal orbitofrontal | 2014 | 1014 |
| **Lateral temporal** | Temporal pole | 2033 | 1033 |
|  | Inferior temporal gyrus | 2009 | 1009 |
|  | Fusiform gyrus | 2007 | 1007 |
|  | Middle temporal gyrus | 2015 | 1015 |
|  | Banks of the superior temporal sulcus | 2001 | 1001 |
|  | Superior temporal gyrus | 2030 | 1030 |
|  | Transverse temporal gyrus | 2034 | 1034 |
| **Pericentral** | Paracentral gyrus | 2017 | 1017 |
|  | Postcentral gyrus | 2022 | 1022 |
|  | Precentral gyrus | 2024 | 1024 |
| **Lateral parietal** | Superior parietal lobule | 2029 | 1029 |
|  | Inferior parietal lobule | 2008 | 1008 |
|  | Supramarginal gyrus | 2031 | 1031 |
| **Lateral Occipital** | Lateral occipital cortex | 2011 | 1011 |
| **Medial Occipital** | Cuneus | 2005 | 1005 |
|  | Pericalcarine cortex | 2021 | 1021 |
|  | Lingual gyrus | 2013 | 1013 |

Label names and numbers are from the Freesurfer 6.0 Desikan-Killiany atlas and color look up table.

Supplemental Table 8. Mean Absolute ± Standard deviation (SD) uptake error (%) compared to Vicra for simulated ^18^F-FDG (60-90 minutes) for the HRRT scanner across 15 subjects.

| **ROI** | **NMC** | **FIR1** | **FIR2** | **1DCOD** | **3DCOD** |
| --- | --- | --- | --- | --- | --- |
| Amygdala | 24.7±11.1 | 3.6±2.9 | 1.4±1.2 | 1.5±1.3 | 1.4±1.1 |
| Caudate | 31.6±13.6 | 9.5±5.1 | 7.7±5.6 | 3.0±1.7 | 2.8±1.9 |
| Cerebellum | 18.8±12.1 | 9.1±3.6 | 1.7±2.0 | 0.7±0.6 | 0.6±0.5 |
| Frontal | 42.9±9.5 | 24.9±3.3 | 12.3±5.6 | 6.1±2.4 | 5.4±2.1 |
| Hippocampus | 14.1±5.4 | 4.6±3.4 | 3.3±2.7 | 1.4±0.8 | 1.1±0.7 |
| Insula | 14.6±6.4 | 4.8±2.8 | 4.3±2.5 | 1.6±0.8 | 1.5±0.8 |
| Occipital | 24.0±7.1 | 20.2±4.9 | 6.9±4.0 | 4.0±2.1 | 4.0±1.9 |
| Parietal | 34.5±10.7 | 24.5±3.3 | 9.5±4.6 | 5.5±2.2 | 5.4±2.0 |
| Putamen | 34.0±11.9 | 16.2±3.2 | 6.0±4.3 | 2.4±1.4 | 1.9±1.3 |
| Temporal | 30.2±9.6 | 19.4±3.3 | 7.3±3.8 | 3.2±1.4 | 3.1±1.3 |
| Thalamus | 22.2±9.6 | 10.9±2.3 | 3.2±2.6 | 1.4±0.8 | 1.5±0.8 |
| **Ave difference (%)** | 26.5 | 13.4 | 5.8 | 2.8 | 2.6 |
| **Ave SD (%)** | 9.7 | 3.5 | 3.5 | 1.4 | 1.3 |

Supplemental Table 9. Mean Absolute ± Standard deviation (SD) uptake error (%) compared to Vicra for simulated ^11^C-RAC (30-60 minutes) for HRRT scanner across 15 subjects.

| **ROI** | **NMC** | **FIR1** | **FIR2** | **1DCOD** | **3DCOD** |
| --- | --- | --- | --- | --- | --- |
| Caudate | 35.2±15.1 | 17.6±5.2 | 9.1±5.4 | 5.8±3.3 | 3.9±2.4 |
| Putamen | 33.8±17.0 | 14.6±4.0 | 6.9±3.6 | 4.4±2.0 | 2.9±1.1 |
| **Ave difference (%)** | 34.5 | 16.1 | 8.0 | 5.1 | 3.4 |
| **Ave SD (%)** | 16.1 | 4.6 | 4.5 | 2.7 | 1.7 |

Supplemental Table 10. Mean ± Standard deviation (SD) uptake error (%) compared to Vicra (ground truth) for the simulated mCT ^18^F-FDG (60-90 minutes) studies across 15 subjects.

| **ROI** | **NMC** | **FIR1** | **FIR2** | **1DCOD** | **3DCOD** |
| --- | --- | --- | --- | --- | --- |
| Amygdala | -18.3±22.4 | -5.1±4.9 | -2.1±4.1 | **0.5±2.5** | 0.1±3.9 |
| Caudate | -38.9±13.4 | -13.2±6.2 | -8.4±6.4 | **-1.5±2.3** | -2.1±2.6 |
| Cerebellum | -20.2±12.7 | -4.5±4.2 | -1.8±1.7 | **-0.6±0.6** | -0.6±0.5 |
| Frontal | -55.6±9.8 | -15.3±5.2 | -11.5±6.3 | **-4.9±2.7** | -4.5±2.1 |
| Hippocampus | -17.0±8.9 | -6.5±4.6 | -3.8±3.6 | **1.1±3.2** | -1.2±1.8 |
| Insula | -14.2±8.5 | -5.1±2.2 | -3.1±2.6 | **-1.0±1.8** | -0.9±1.4 |
| Occipital | -23.8±7.5 | -10.4±5.9 | -6.5±3.9 | **-4.4±2.6** | -3.8±2.5 |
| Parietal | -36.0±11.2 | -14.7±5.0 | -9.1±5.3 | **-4.6±3.4** | -4.6±2.8 |
| Putamen | -38.1±13.1 | -8.8±5.1 | -6.2±5.4 | **-1.1±1.4** | -1.2±1.6 |
| Temporal | -30.8±9.4 | -10.4±5.0 | -7.0±3.9 | **-2.4±1.8** | -2.2±1.7 |
| Thalamus | -25.3±11.2 | -5.8±3.2 | -3.9±2.6 | **-1.0±0.9** | -1.1±1.0 |
| **Ave difference (%)** | -27.9 | -16.8 | -5.8 | -2.0 | -2.0 |
| **Ave SD (%)** | 11.5 | 3.5 | 4.2 | 2.1 | 2.0 |

Supplemental Table 11. Mean ± Standard deviation (SD) uptake error (%) compared to Vicra (ground truth) for the simulated mCT ^11^C-RAC (30-60 minutes) studies across 15 subjects.

| ROI | NMC | FIR1 | FIR2 | 1DCOD | 3DCOD |
| --- | --- | --- | --- | --- | --- |
| Caudate | -39.9±16.1 | -17.2±4.4 | -9.7±5.6 | 2.9±3.0 | -3.3±2.7 |
| Putamen | -34.4±17.8 | -12.3±3.3 | -6.6±4.1 | 3.3±2.5 | -2.6±1.7 |
| **Ave difference (%)** | -37.2 | -14.8 | -8.2 | 3.1 | -3.0 |
| **Ave SD (%)** | 16.9 | 3.9 | 4.9 | 2.8 | 2.2 |

**Supplemental Table 12.** Mean Absolute ± Standard deviation (SD) uptake error (%) compared to Vicra for real ^18^F-FDG (60-90 minutes) for the HRRT scanner across 10 subjects.

| **ROI** | **NMC** | **FIR1** | **FIR2** | **1DCOD** | **3DCOD** |
| --- | --- | --- | --- | --- | --- |
| Amygdala | 12.1±4.9 | 4.0±2.9 | 2.0±1.2 | 4.1±5.4 | 3.0±1.3 |
| Caudate | 23.5±10.6 | 7.9±4.6 | 6.1±3.3 | 7.5±5.6 | 3.7±3.2 |
| Cerebellum | 13.4±8.2 | 4.3±3.3 | 1.6±2.0 | 1.8±1.9 | 1.4±1.6 |
| Frontal | 22.9±9.1 | 5.5±4.3 | 4.4±1.9 | 4.2±3.8 | 2.8±2.1 |
| Hippocampus | 9.0±4.6 | 4.0±2.8 | 2.5±1.5 | 4.4±2.1 | 2.9±2.1 |
| Insula | 7.7±5.5 | 3.2±2.5 | 1.0±0.5 | 1.8±1.1 | 1.6±0.9 |
| Occipital | 13.7±5.1 | 5.6±5.9 | 4.4±6.9 | 3.5±5.6 | 3.1±4.6 |
| Parietal | 17.4±9.5 | 5.9±4.0 | 3.8±2.1 | 3.9±3.4 | 2.3±1.2 |
| Putamen | 20.9±8.7 | 4.9±3.0 | 3.0±2.9 | 2.7±3.3 | 1.4±0.7 |
| Temporal | 18.4±7.7 | 5.0±2.3 | 2.6±1.8 | 2.4±2.8 | 2.2±2.2 |
| Thalamus | 15.4±8.7 | 5.5±3.4 | 2.0±1.3 | 3.3±3.5 | 1.6±0.8 |
| **Ave difference (%)** | 15.9 | 5.0 | 3.0 | 3.6 | 2.4 |
| **Ave SD (%)** | 7.5 | 3.4 | 2.2 | 3.5 | 1.9 |

Supplemental Table 13. Mean Absolute ± Standard deviation (SD) uptake error (%) compared to Vicra for real ^11^C-RAC (30-60 minutes) for HRRT scanner across 10 subjects.

| **ROI** | **NMC** | **FIR1** | **FIR2** | **1DCOD** | **3DCOD** |
| --- | --- | --- | --- | --- | --- |
| Caudate | 28.1±14.2 | 15.4±13.7 | 8.5±5.5 | 4.6±4.0 | 3.0±2.3 |
| Putamen | 20.8±14.9 | 11.4±14.8 | 5.2±4.5 | 2.1±2.6 | 1.8±2.4 |
| **Ave difference (%)** | 24.5 | 13.4 | 6.9 | 3.3 | 2.4 |
| **Ave SD (%)** | 14.6 | 14.3 | 5.0 | 3.3 | 2.4 |

Supplemental Table 14. Mean uptake error (%) compared to Vicra for the real ^11^C-MRB (0-90 minutes) study performed on the mCT.

| **ROI** | **NMC** | **3DCOD** |
| --- | --- | --- |
| Caudate | 12.6 | 5.6 |
| Cerebellum Cortex | -15.2 | 0.5 |
| Putamen | -7.9 | 1.6 |
| Thalamus | -9.8 | 0.6 |
| **Ave difference (%)** | -5.1 | 2.1 |
| **Ave SD (%)** | 12.2 | 2.4 |

Supplemental Table 15. Mean uptake error (%) compared to Vicra for the real ^11^C-PBR28 (0-90 minutes) study on the mCT.

| **ROI** | **NMC** | **3DCOD** |
| --- | --- | --- |
| Caudate | -5.2 | 4.8 |
| Putamen | 2.7 | 0.4 |
| Thalamus | -11.0 | 3.0 |
| **Ave difference (%)** | -4.5 | 2.7 |
| **Ave SD (%)** | 6.9 | 2.2 |

**Supplemental Table 16.** Recommended default values for different parameters used in the proposed motion detection algorithm for a 90-min study.

| **Parameter** | **Default Value** |
| --- | --- |
| *n*_max_ | 300 |
| *α* | 1.0 (^18^F-FDG); 1.6 (^11^C-RAC) |
| *s* | 6 |
| *P* | 3 |

Supplemental Table 17. Mean absolute uptake error (%) compared to Vicra for the real ^18^F-FDG (60-90 minutes) study for 3DCOD and COD*.

| **ROI** | **NMC** | **COD*** | **3DCOD** |
| --- | --- | --- | --- |
| Amygdala | 13.4 | 8.1 | 3.9 |
| Caudate | 21.2 | 12.2 | 2.2 |
| Cerebellum | 14.2 | 3.3 | 0.2 |
| Frontal | 16.3 | 0.7 | 1.1 |
| Hippocampus | 5.3 | 6.7 | 2.6 |
| Insula | 5.8 | 5.9 | 0.0 |
| Occipital | 19.7 | 3.2 | 1.6 |
| Parietal | 12.4 | 0.3 | 3.4 |
| Putamen | 19.0 | 0.4 | 2.8 |
| Temporal | 18.4 | 2.4 | 1.0 |
| Thalamus | 11.9 | 5.9 | 2.6 |
| **Ave difference (%)** | 14.3 | 4.5 | 1.9 |
| **Ave SD (%)** | 5.3 | 3.7 | 1.3 |


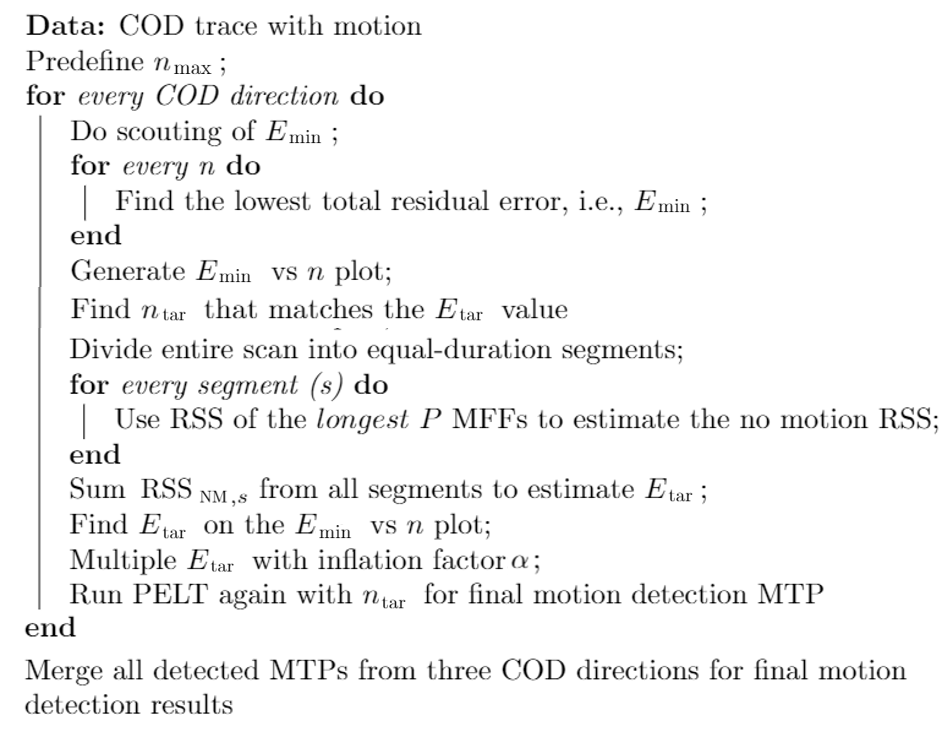


Supplemental Figure 1. Pseudo code of the proposed adaptive data-driven motion detection algorithm.


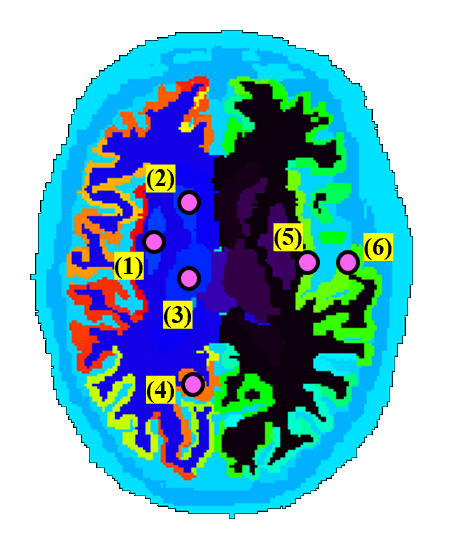


Supplemental Figure 2. Example of a FreeSurfer segmented brain. Pink dots indicate a few examples of the center of mass (COM) for different brain regions: (1) right putamen, (2) caudate, (3) thalamus proper, (4) precuneus, (5) left insula and a portion of (6) left superior temporal cortex.


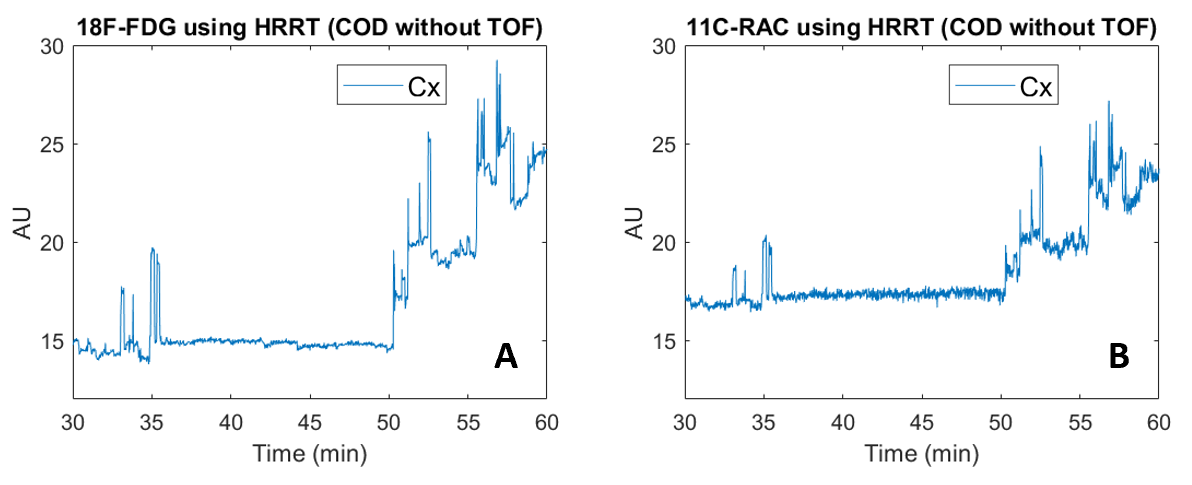


Supplemental Figure 3. COD trace in lateral direction (*C*_x_) for a simulated (A) ^18^F-FDG and (B) ^11^C-RAC in HRRT using the same subject and same motion information. AU: Arbitrary Unit.


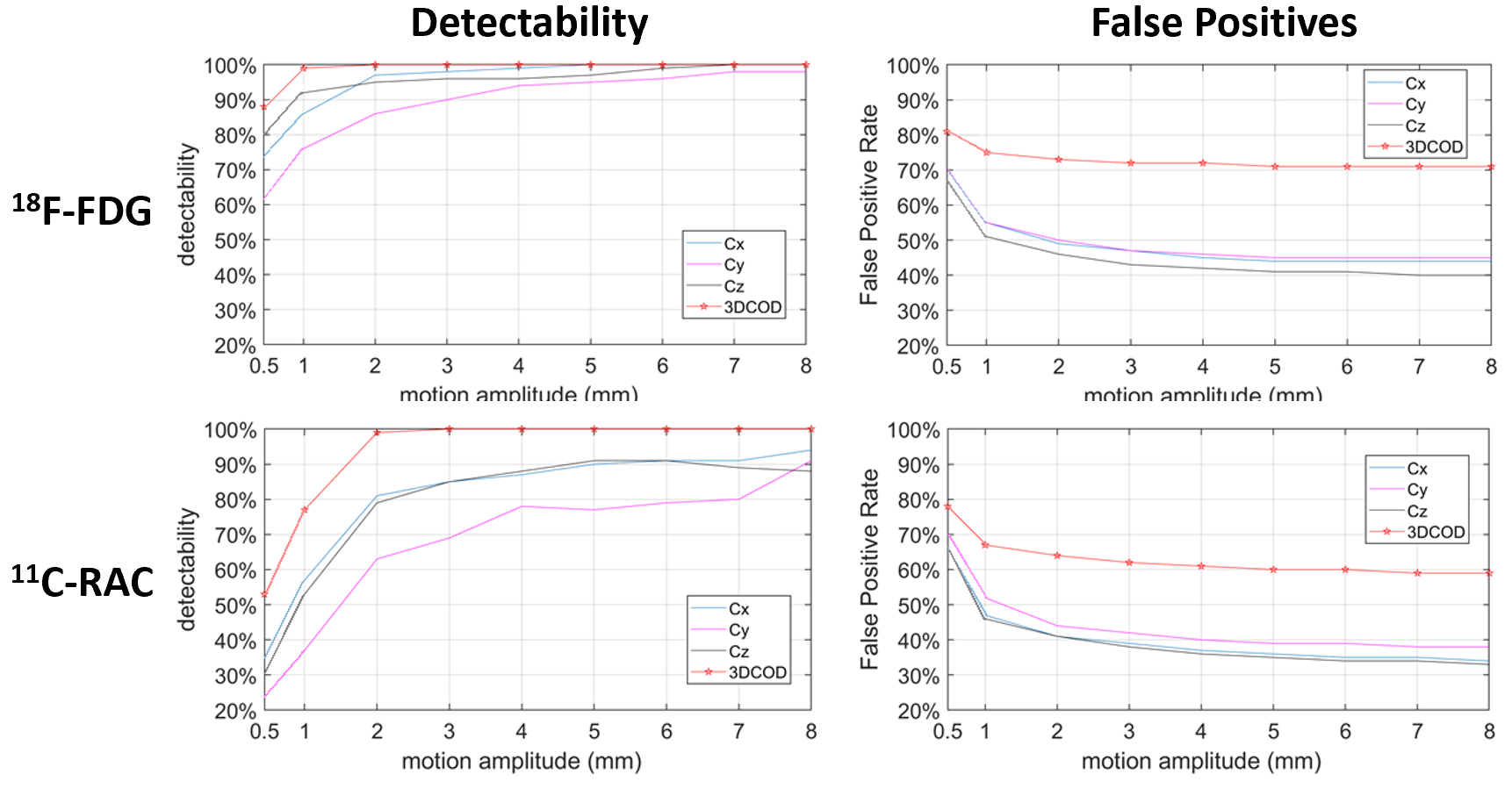


**Supplemental Figure 4.** Detectability and false positive rate results for 3DCOD as a function of α for ^18^F-FDG (top row) and ^11^C-RAC (bottom row) simulated for the mCT scanner. Red line with stars indicates the 3DCOD method. Inflation factor *α* was set to 1.0 and 1.6 for ^18^F-FDG and ^11^C-RAC studies, respectively.


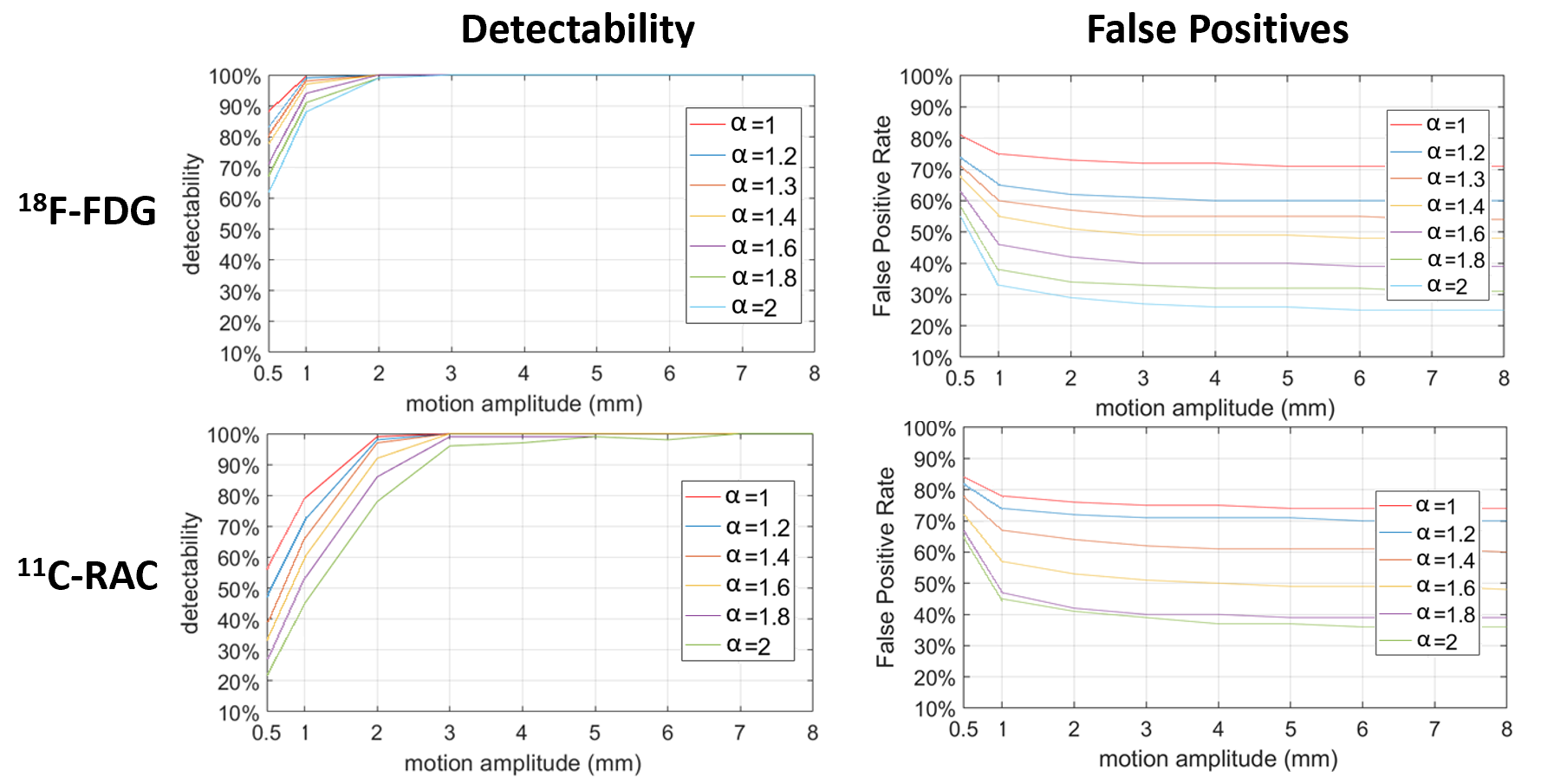


**Supplemental Figure 5.** Detectability and false positive rate results for 3DCOD as a function of α for ^18^F-FDG (top row) and ^11^C-RAC (bottom row) simulated for the mCT scanner.


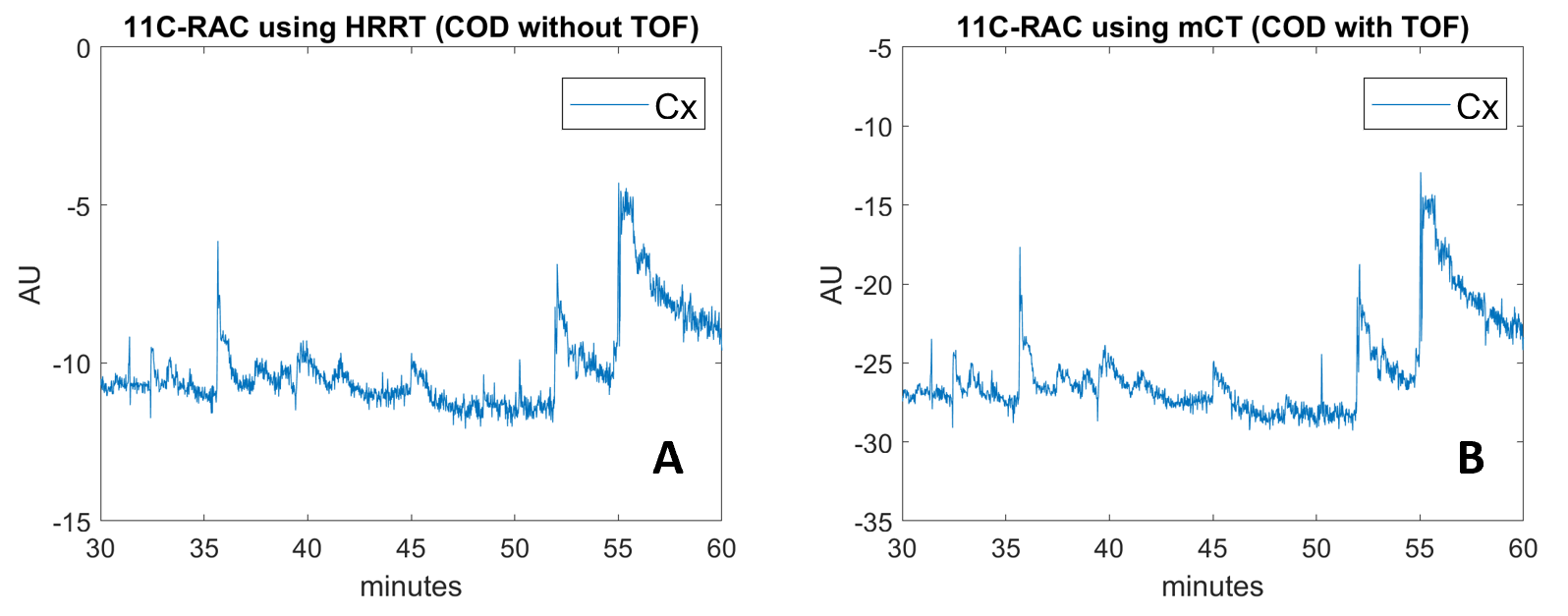


Supplemental Figure 6. COD trace in lateral direction (*C_x_*) for a simulated ^11^C-RAC in (A) HRRT (COD without TOF) and (B) mCT (COD with TOF) using the same brain and same motion information. mCT yielded less photon noise on the COD than HRRT.


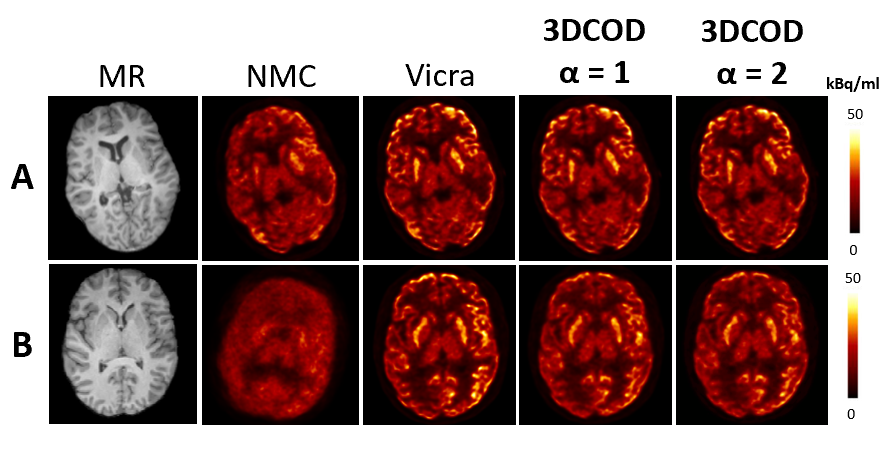


**Supplemental Figure 7.** Sample slices of motion-corrected reconstructions of simulated HRRT ^18^F-FDG study (60-90 min) showing different inflation factor *α*. Studies from (A) and (B) are ranked 1/15 (best case) and 15/15 (worst) based on mean difference for the 3DCOD-based approach, respectively.


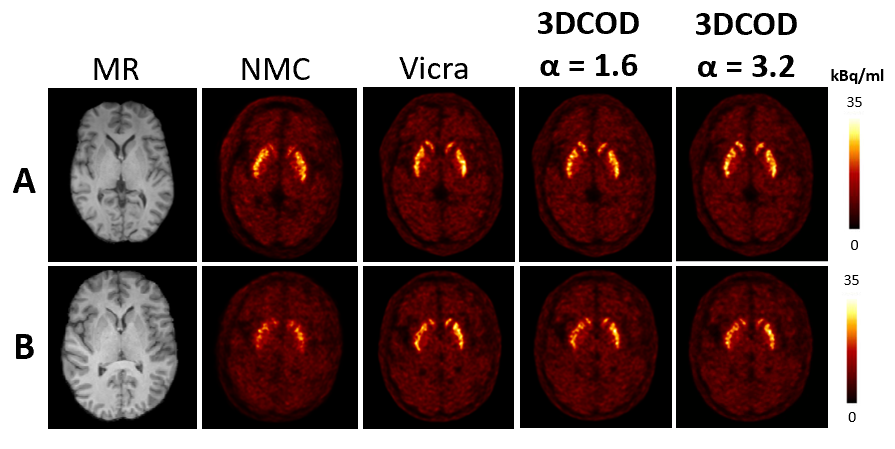


**Supplemental Figure 8.** Sample slices of motion-corrected reconstructions of simulated HRRT ^11^C-RAC study (30-60 min) showing different inflation factor *α*. Studies from (A) and (B) are ranked 1/15 (best case) and 15/15 (worst) based on mean difference for the 3DCOD-based approach, respectively.


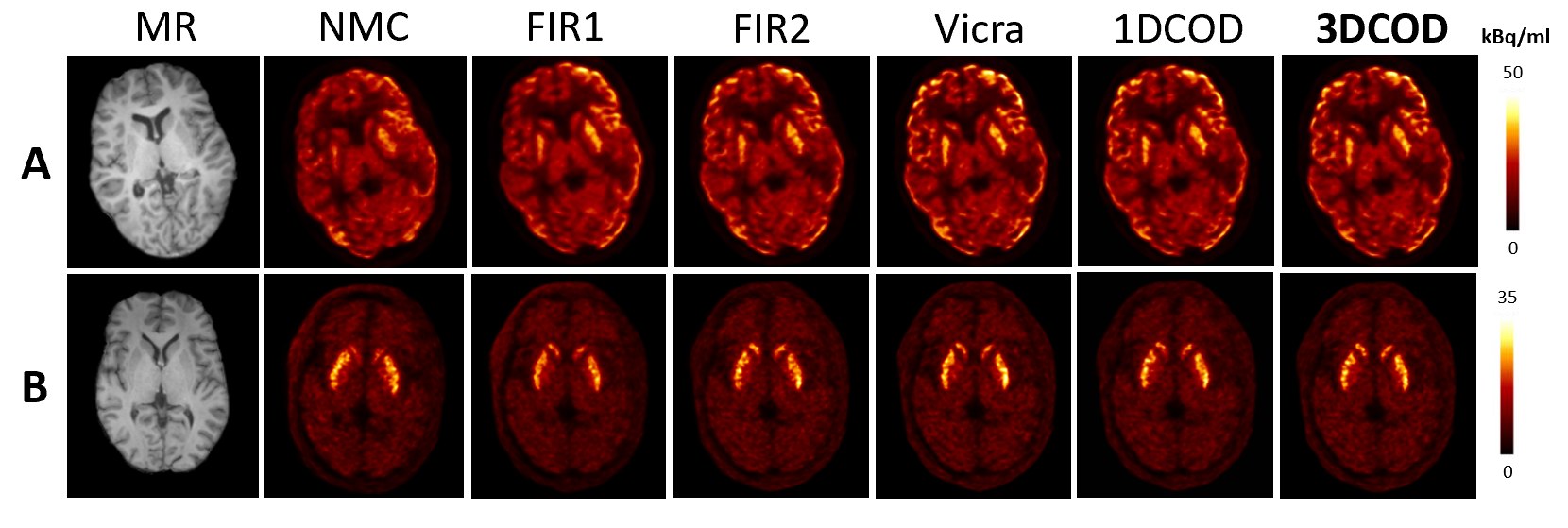


**Supplemental Figure 9.** Sample slices of motion-corrected reconstructions of simulated HRRT (A) ^18^F-FDG study (60-90 min) and (B) ^11^C-RAC study (30-60 min). Studies are ranked 1/15 (best case) based on mean difference for the 3DCOD-based approach.


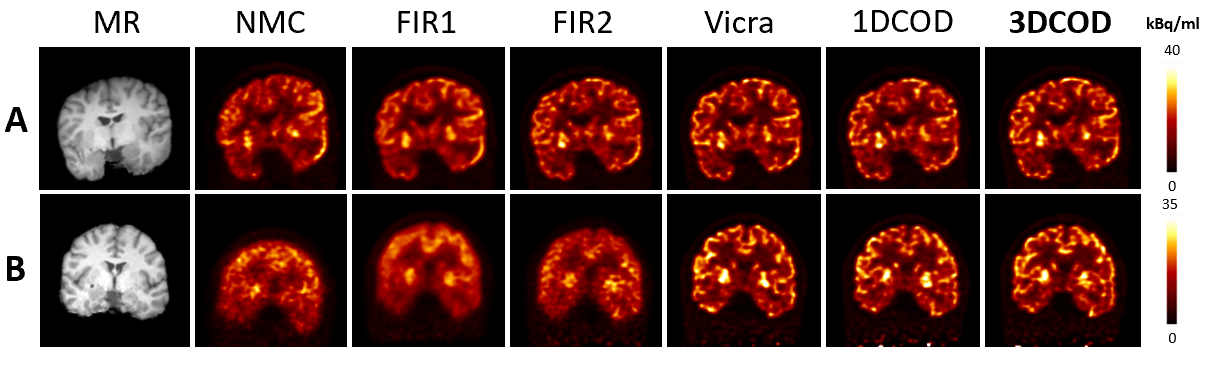


Supplemental Figure 10. Sample slices of motion-corrected reconstructions of simulated mCT ^18^F-FDG studies (60-90 min). Studies from (A) and (B) are ranked 1/15 (best case) and 15/15 (worst) of the 3DCOD-based approach, respectively.


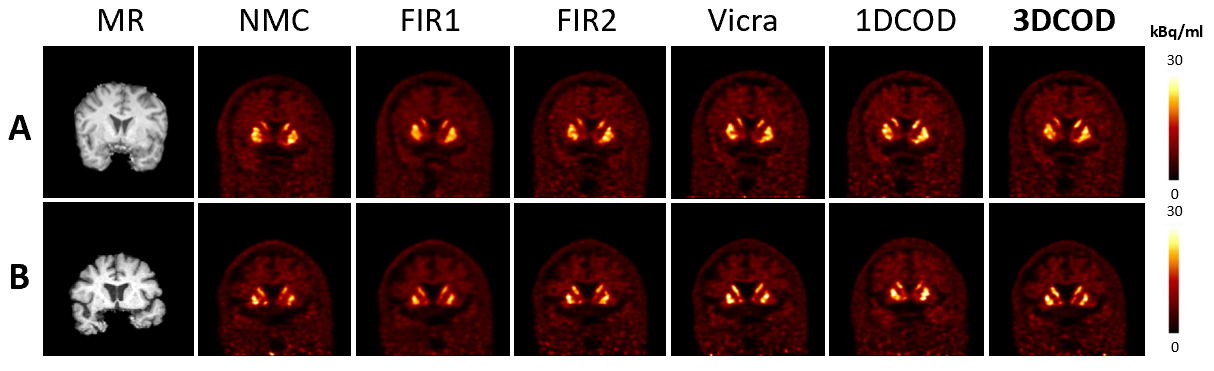


Supplemental Figure 11. Sample slices of motion-corrected reconstructions of simulated mCT ^11^C-RAC studies (30-60 min). Studies from (A) and (B) are ranked 1/15 (best case) and 15/15 (worst) of the 3DCOD-based approach, respectively.


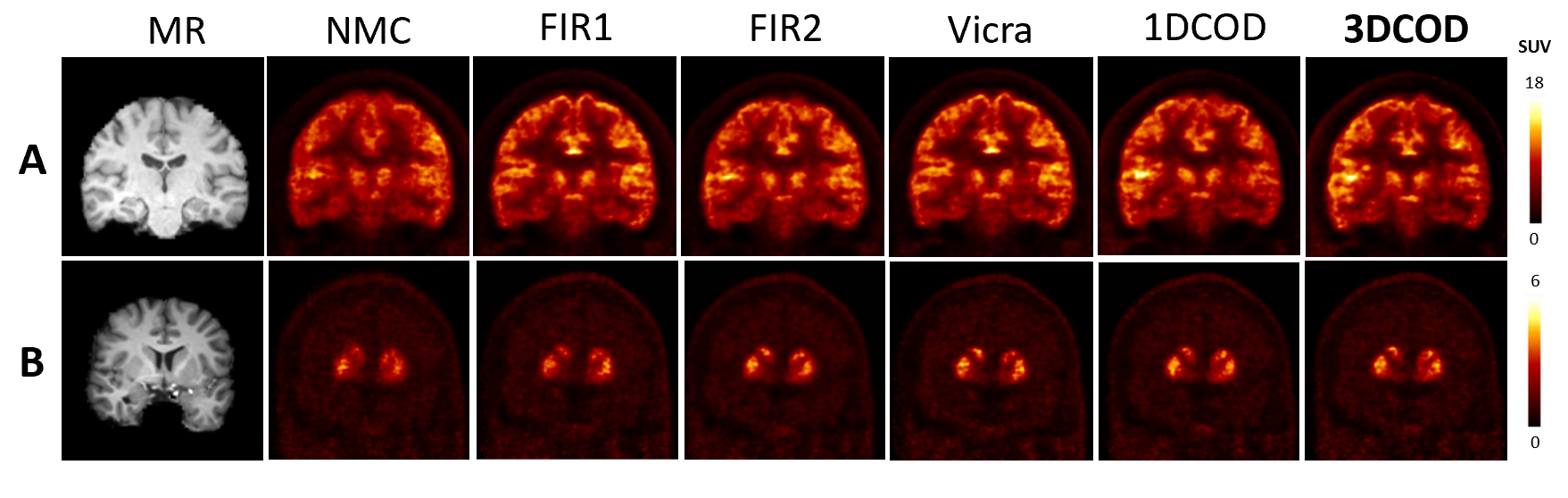


**Supplemental Figure 12.** Sample slices of motion-corrected reconstructions of real (A) ^18^F-FDG (60-90 min) and (B) ^11^C-RAC (30-60 min) studies. Studies are ranked 1/10 (best case) based on mean difference for the 3DCOD-based approach.


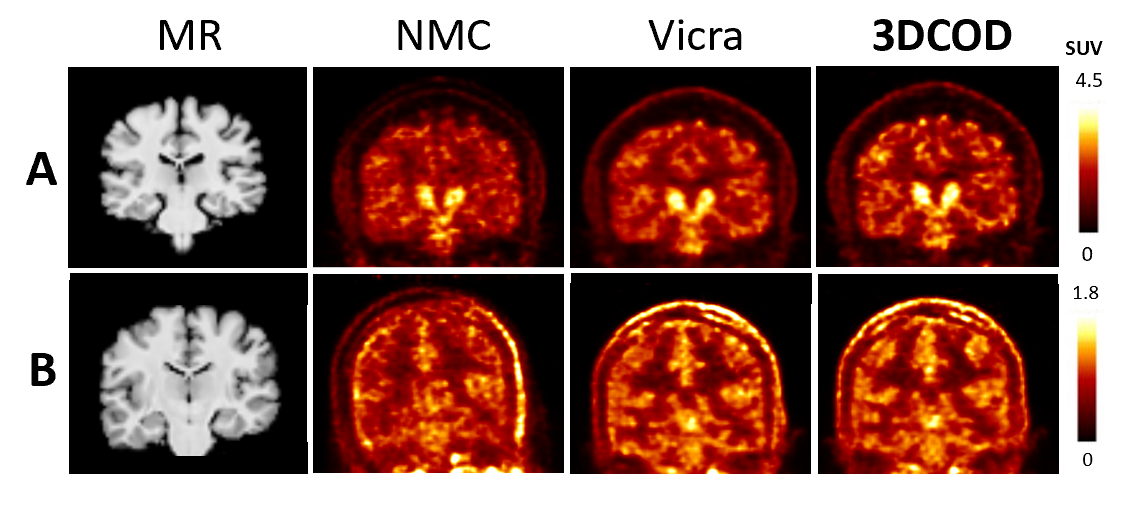


Supplemental Figure 13. Motion-corrected reconstructions of a (A) 60-90 min ^11^C-MRB and (B) 0-90 min ^11^C-PBR28 studies performed on the mCT.


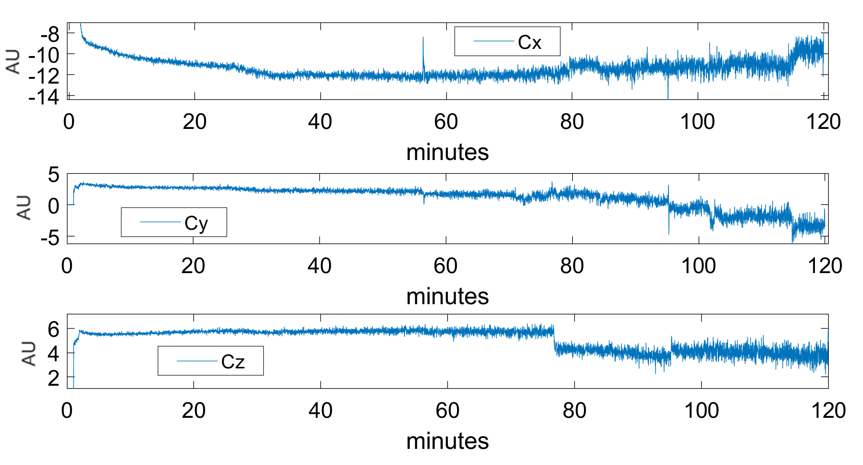


Supplemental Figure 14. COD traces in all three directions for the real ^11^C-MRB study. Large motion is evident starting 75 min post injection in the *z* direction.


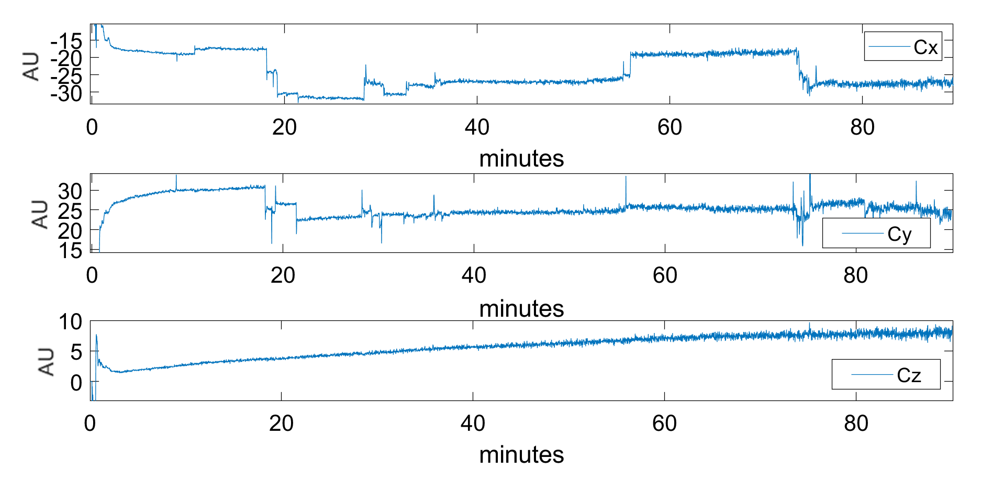


Supplemental Figure 15. COD traces in all three directions for the real ^11^C-PRB28 mCT study.


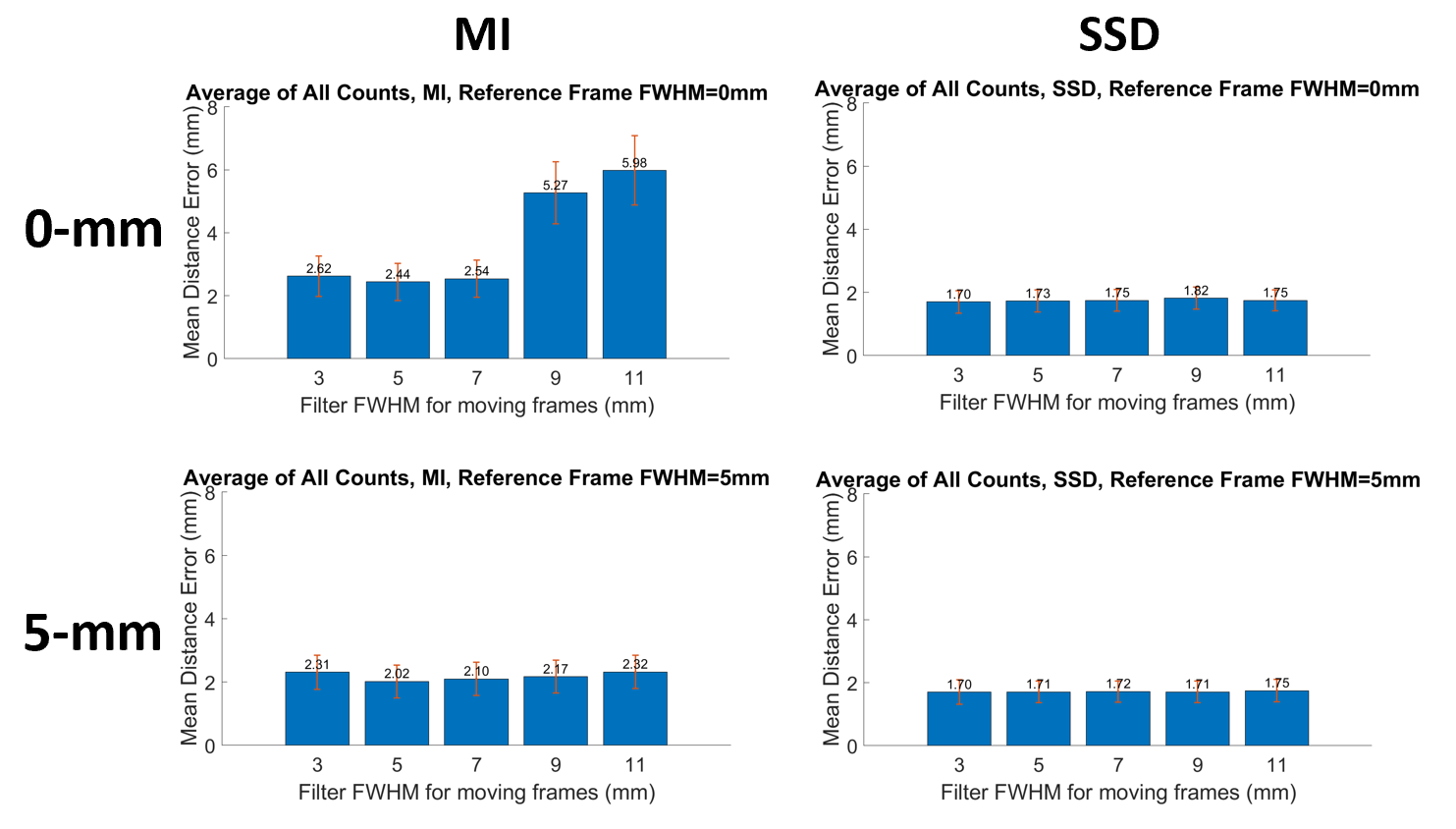


Supplemental Figure 16. Mean distance error (MDE) results for simulated ^18^F-FDG in HRRT scanner, comparing sum of squared difference (SSD) and mutual information (MI) with different filter FWHM applied to reference and moving frames.


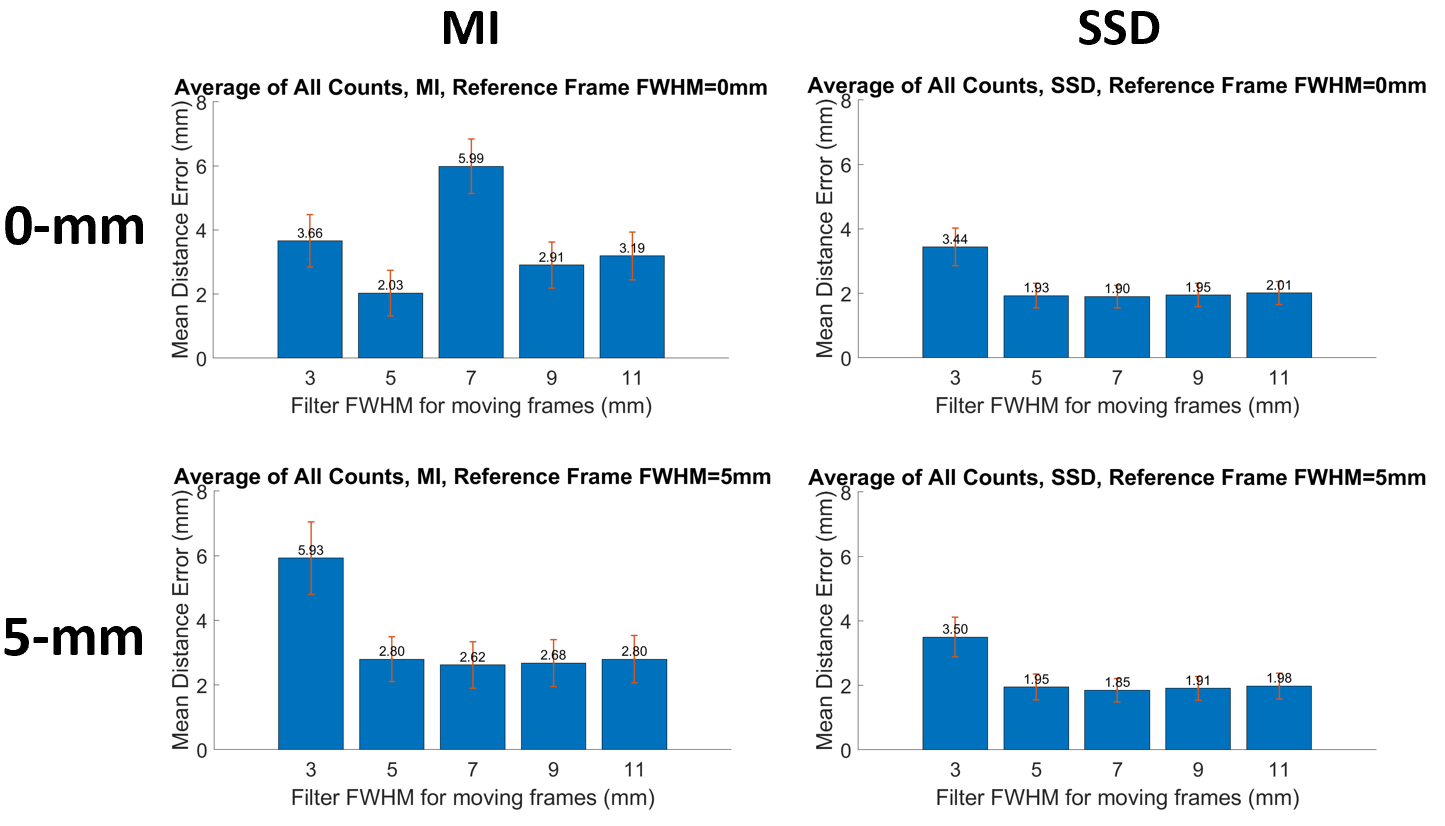


Supplemental Figure 17. Mean distance error (MDE) results for simulated ^18^F-FDG in mCT, comparing sum of squared difference (SSD) and mutual information (MI) with different filter FWHM applied to reference and moving frames. The high MDE for the 7-mm moving frame filter FHWM in MI was due to an outlier in the data.


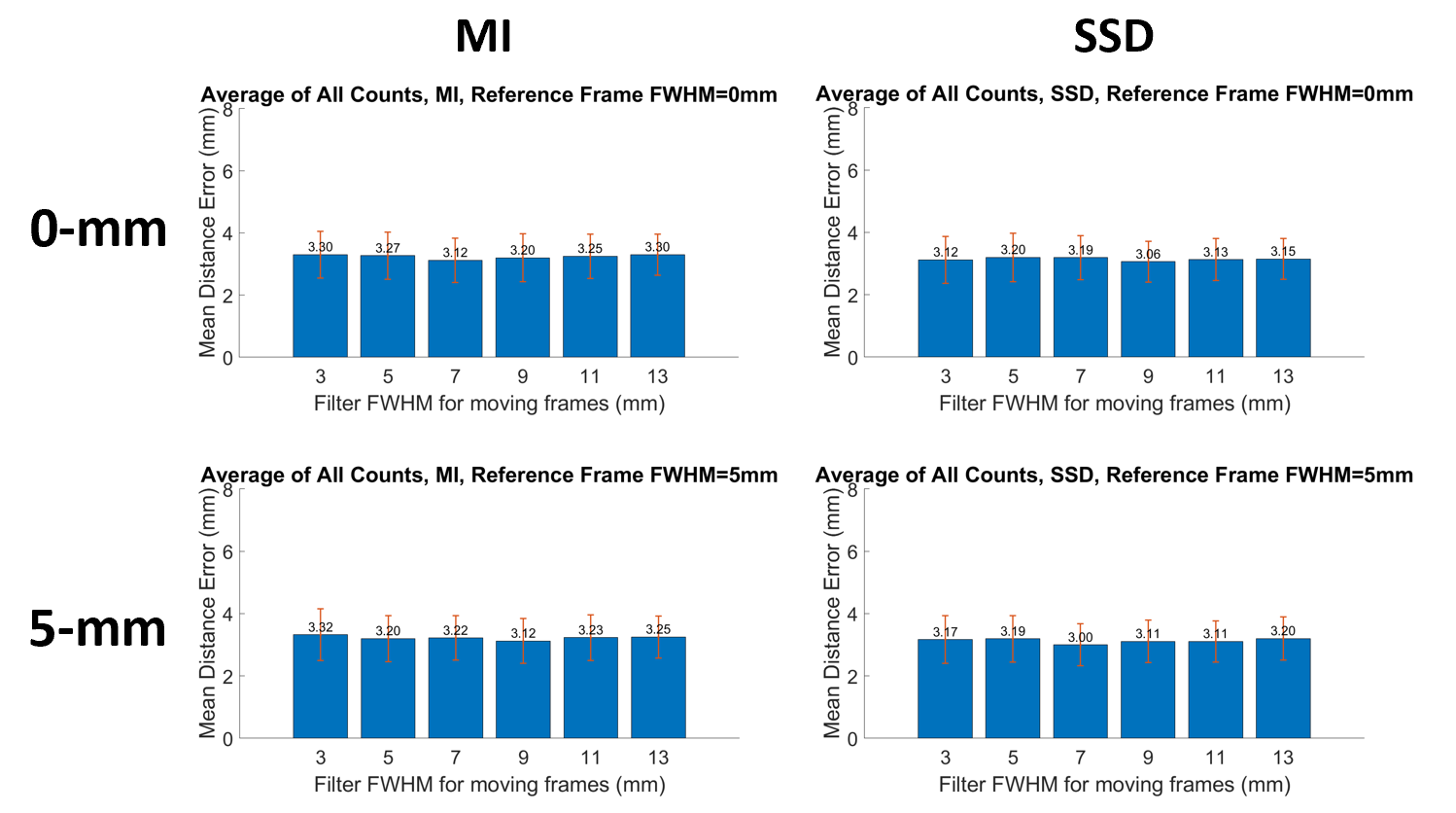


Supplemental Figure 18. Mean distance error (MDE) results for simulated ^11^C-RAC in HRRT, comparing sum of squared difference (SSD) and mutual information (MI) with different filter FWHM applied to reference and moving frames.


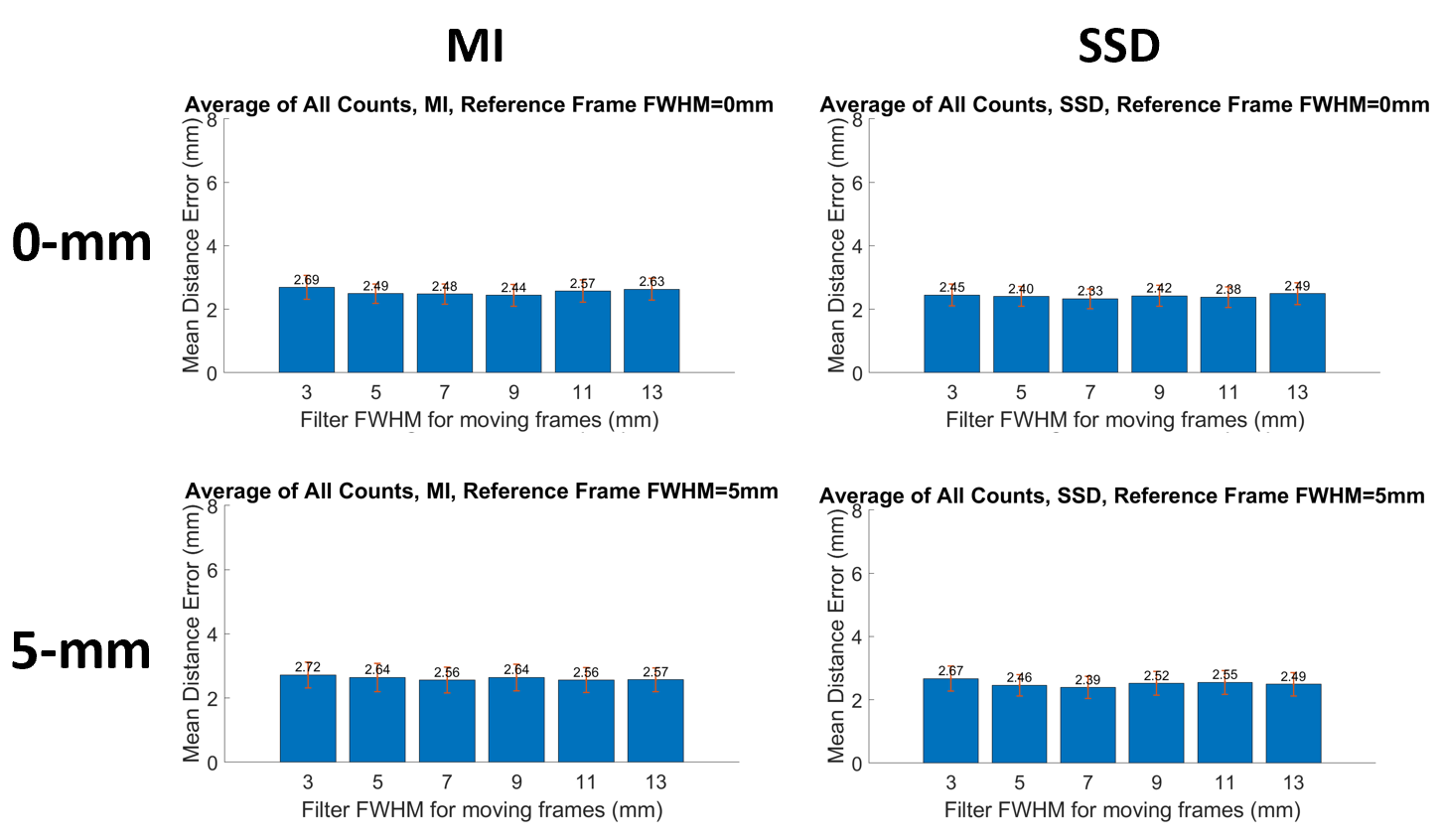


Supplemental Figure 19. Mean distance error (MDE) results for simulated ^11^C-RAC in mCT, comparing sum of squared difference (SSD) and mutual information (MI) with different filter FWHM applied to reference and moving frames.


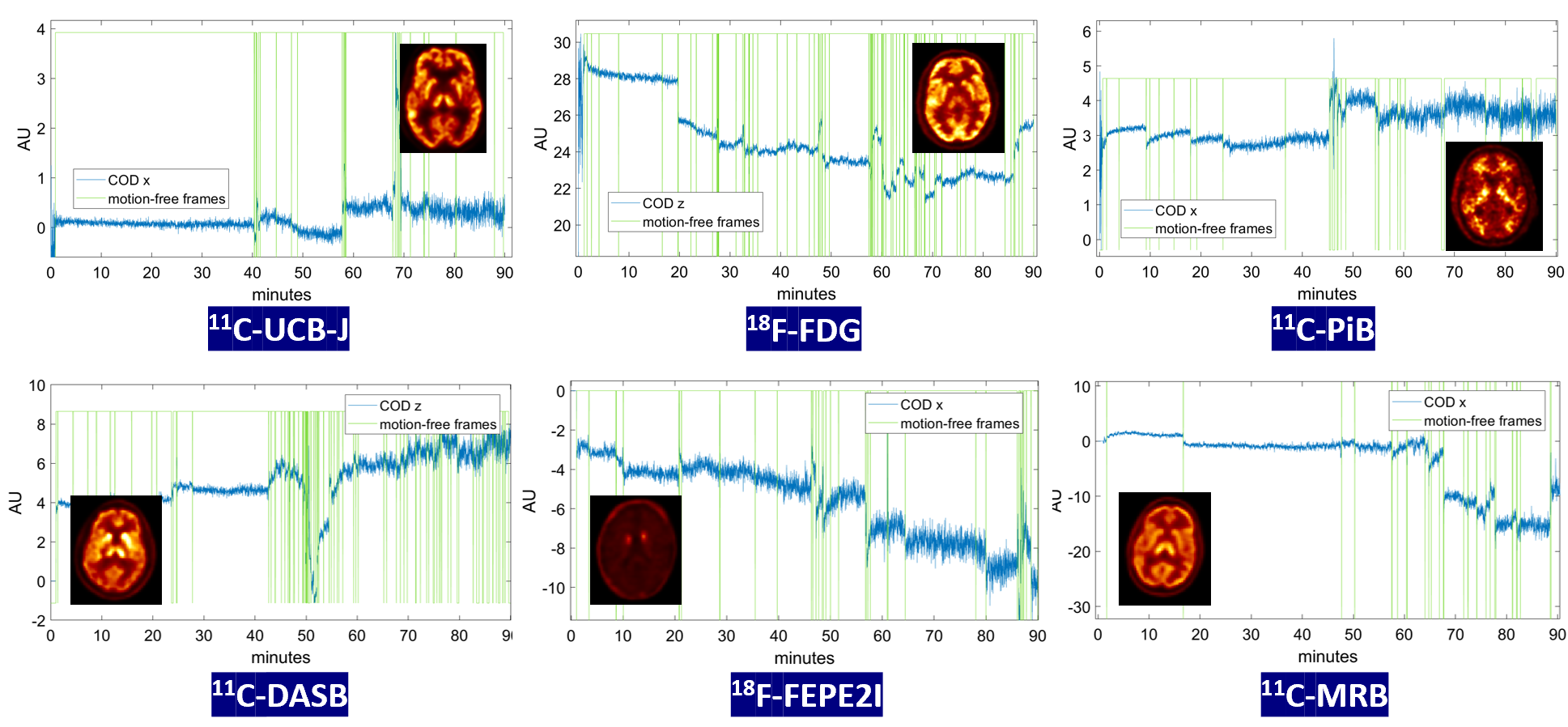


Supplemental Figure 20. Examples of motion detection without parameter tuning using different tracers.
